# Synergistic coupling between 3D bioprinting and vascularization strategies

**DOI:** 10.1088/1758-5090/ad0b3f

**Published:** 2023-11-20

**Authors:** Miji Yeo, Anwita Sarkar, Yogendra Pratap Singh, Irem Deniz Derman, Pallab Datta, Ibrahim T Ozbolat

**Affiliations:** 1 The Huck Institutes of the Life Sciences, Penn State University, University Park, PA 16802, United States of America; 2 Engineering Science and Mechanics Department, Penn State University, University Park, PA 16802, United States of America; 3 Department of Pharmaceutics, National Institute of Pharmaceutical Education and Research, Kolkata, West Bengal 700054, India; 4 Department of Biomedical Engineering, Penn State University, University Park, PA 16802, United States of America; 5 Materials Research Institute, Penn State University, University Park, PA 16802, United States of America; 6 Department of Neurosurgery, Penn State College of Medicine, Hershey, PA 17033, United States of America; 7 Penn State Cancer Institute, Penn State University, Hershey, PA 17033, United States of America; 8 Biotechnology Research and Application Center, Cukurova University, Adana 01130, Turkey

**Keywords:** Bioprinting, tissue and organ substitutes, vascularization, vascularized tissues, intraoperative bioprinting

## Abstract

Three-dimensional (3D) bioprinting offers promising solutions to the complex challenge of vascularization in biofabrication, thereby enhancing the prospects for clinical translation of engineered tissues and organs. While existing reviews have touched upon 3D bioprinting in vascularized tissue contexts, the current review offers a more holistic perspective, encompassing recent technical advancements and spanning the entire multistage bioprinting process, with a particular emphasis on vascularization. The synergy between 3D bioprinting and vascularization strategies is crucial, as 3D bioprinting can enable the creation of personalized, tissue-specific vascular network while the vascularization enhances tissue viability and function. The review starts by providing a comprehensive overview of the entire bioprinting process, spanning from pre-bioprinting stages to post-printing processing, including perfusion and maturation. Next, recent advancements in vascularization strategies that can be seamlessly integrated with bioprinting are discussed. Further, tissue-specific examples illustrating how these vascularization approaches are customized for diverse anatomical tissues towards enhancing clinical relevance are discussed. Finally, the underexplored intraoperative bioprinting (IOB) was highlighted, which enables the direct reconstruction of tissues within defect sites, stressing on the possible synergy shaped by combining IOB with vascularization strategies for improved regeneration.

## 1. Introduction

Significant advancements in the realm of three-dimensional (3D) bioprinting in the past decade have raised the promise of alleviating the rapidly growing crisis of donor organ shortage for transplantation procedures. Bioprinting involves the precise deposition of biological elements, such as cells and/or other biologics, to construct functional tissue and organ substitutes [1]. Given its ability and potential to fabricate these functional tissue and organ substitutes, bioprinting has found a wide range of applications in the medical and healthcare sector, ranging from drug discovery and disease modeling to regenerative medicine and transplantation. Bioprinting can improve transplantation surgery by diminishing the complexity and morbidity of tissue harvesting and associated use of immunosuppression through the development of patient-specific living implants [2]. The potential application of 3D bioprinting in surgery and personalized healthcare involves the use of patient-specific autologous cellular materials to produce *de novo* organs.

The essential components required to 3D bioprint living tissues and organs usually include autologous and patient-specific cells and biocompatible and degradable bioinks [3]. Stem cells are considered as the ideal cell source as they have the ability to differentiate into multiple cell phenotypes for bioprinting of patient-specific tissue and organ substitutes for transplantation [4]. The bioinks must be cell-friendly, mechanically strong, and affordable, create a conducive environment for cell migration and proliferation, and possess the ability to remain structurally stable upon bioprinting.

3D Bioprinting enables the customization of complex tissue architecture using a combination of materials and printing technologies to create various tissue types, and, eventually, functional replacement organs. Towards its realization, the inclusion of complex vascular networks is essential for tissue survival [5]. Rapid advancements in recent years have taken huge strides toward the incorporation of vascular networks in 3D bioprinted tissues and organs. Recent progress in vascularization have enabled the fabrication of viable, functional tissue and organ substitutes such as but not limited to skin [6], bone [7], heart [8], liver [9] and muscle [10]. For instance, patient-specific skin and skin appendages have been fabricated using different bioprinting techniques with potential use in the treatment of skin ulcers and non-healing cutaneous wounds [6]. Generation of metabolically highly active tissues has offered an approach in healthcare to improve the survival rate and quality of life. However, to achieve anatomically-relevant, functional tissues and organs, it is important to develop a customized tissue-specific bioink with suitable biomaterial, cell source and bioactive factor selections along with the inclusion of penetrating vasculature and neural networks.

To guide future research, a comprehensive review on current developments in synergistic coupling between 3D bioprinting and vascularization strategies is required. Although there are several reviews focusing on 3D bioprinting of vascular [11] and vascularized tissues [12] with tissue-specific examples, this review introduces a more complete view of developing vasculature and vascularized tissues and organs using recent technical advances, and covers from pre- to post-bioprinting processes, including imaging, biomaterial selection, bioprinting modalities, and post-printing perfusion/maturation in the context of vascularization followed by comprehensive tissue-specific and intraoperative applications. The current comprehensive review sets itself apart by amalgamating the latest advances in both 3D bioprinting and vascularization strategies, offering readers a contemporary and holistic perspective on this dynamic interplay. The synergistic coupling between 3D bioprinting and vascularization strategies is of utmost importance as vascularization enhances tissue viability and function by ensuring proper blood supply and bioprinting can enable the creation of highly specialized, tissue-specific vascular networks. Thus, section 2 of this article covers the entire bioprinting process, from pre-bioprinting stages to post-printing processing of constructs *via* perfusion and maturation, ensuring a comprehensive understanding of the topic. Section 3 discusses the advances in recent vascularization strategies that can be coupled with bioprinting. Tissue-specific examples in section 4 shed light on how vascularization strategies are tailored to diverse anatomical tissues, such as skin, muscle, bone, cardiac, and liver tissues, adding clinical relevance. Moreover, the review highlights an exploration of intraoperative bioprinting (IOB) in section 5, a transformative aspect often underrepresented in existing literature.

## 2. Bioprinting and its major stages and modalities

The fundamental principles of bioprinting have been extensively reviewed in the literature [13–17]. Briefly, bioprinting allows the layer-by-layer precise deposition of biologics such as but not limited to cells, proteins, growth factors, drugs, and DNA with or without exogenous biomaterials to fabricate biomimetic functional tissue and organ substitutes [18]. The process of bioprinting tissue and organ substitutes involves multiple steps, which are classified under three major phases, involving pre-bioprinting, bioprinting and post-bioprinting.

### 2.1. Pre-bioprinting

The essential components of the pre-bioprinting stage entail minimally-invasive tissue biopsy to obtain viable cells, efficient protocols to maintain these during culture, high-resolution imaging to acquire structural and anatomic data, and creation of 3D models of the target tissues or organs using various modeling approaches [18]. Prior to bioprinting, designing of injury-specific and biomimetic bioprinted constructs requires the understanding of the anatomy of targeted tissues or organs. In addition, accurate information on an integrated vascular network is highly essential to ensure adequate and proper nutrient supply. Thus, high-resolution medical imaging exhibit great significance in the process of bioprinting [19].

**Figure 1.**
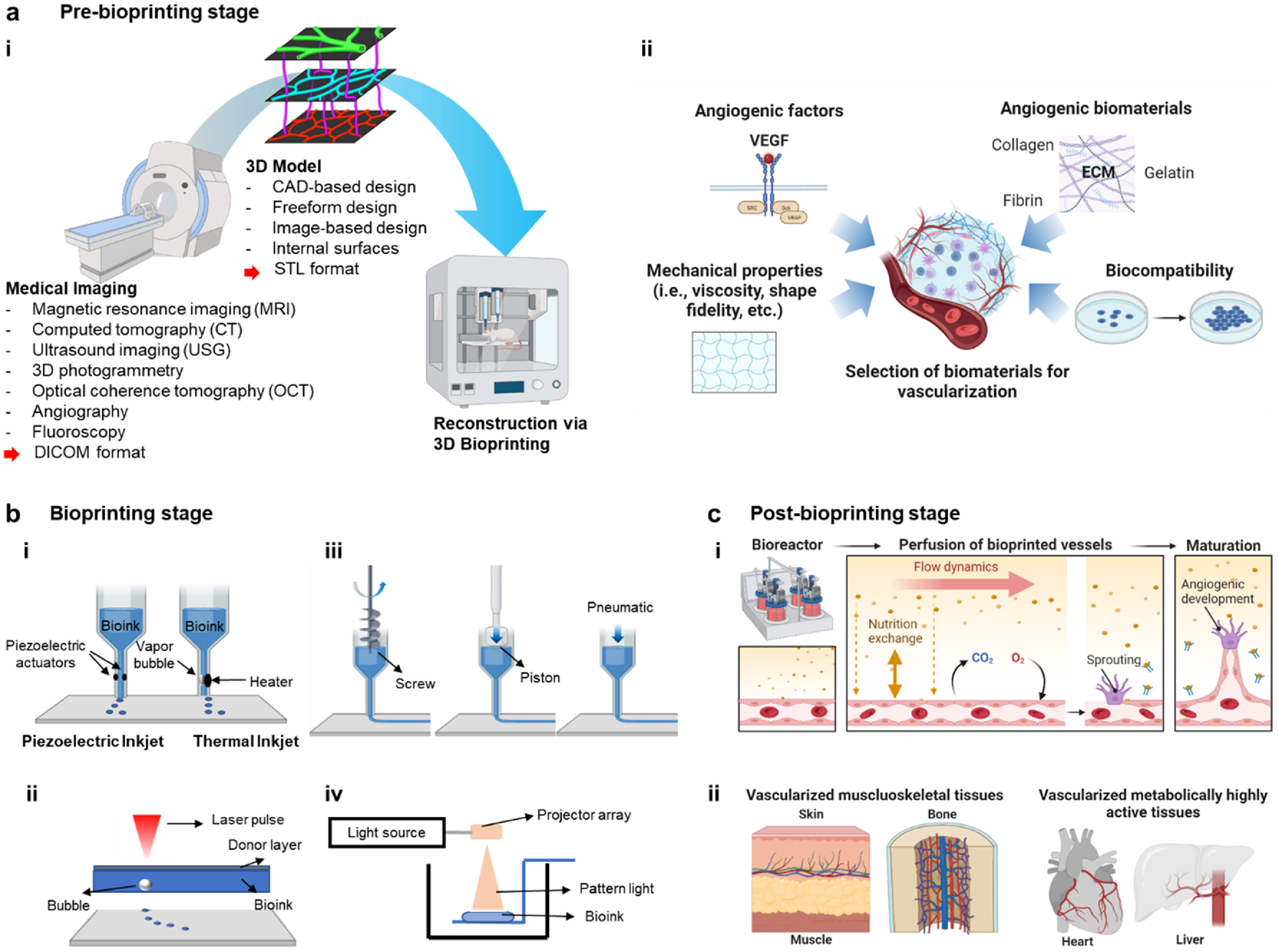
(a) Pre-bioprinting stage. (i) Process of obtaining anatomic data via medical and vascular imaging for 3D bioprinting. (ii) Selection of biomaterials and its relevant criteria for vascularization. (b) Bioprinting stage and common bioprinting techniques: (i) droplet-, (ii) laser-, (iii) extrusion- and (iv) light-based bioprinting. (c) Post-bioprinting stage. (i) Utilization of bioreactor for inducing angiogenic development and enhancing maturation. (ii) Vascularization strategies coupled with bioprinted tissues for *in-vitro* and *in-vivo* applications. This figure was created with BioRender.com.

Currently, magnetic resonance imaging (MRI), computed tomography (CT), ultrasound imaging (USG) and various other imaging modalities (i.e., optical coherence tomography (OCT), angiography, etc [20]) are used to obtain 3D anatomies of tissues and organs (figure 1(a)). MRI is a highly preferred modality for imaging soft tissues, which utilizes pulsed radiofrequency electromagnetic waves to visualize the target [21]. CT provides high-resolution images compared to MRI images, with reduced time of scan. Anatomy can be conveniently obtained from CT images, which involves radiation. Further, the advancement from CT to micro-CT enables to characterize not only bulk anatomy but also the microstructural and mechanical properties of scaffolds, which is widely used for imaging bone density alteration and regeneration of the tissue. On the other hand, UGS is considered the safest imaging modality and has recently been suggested as a way to give bioprinted part sub-surface information. OCT is widely utilized in the disciplines of biological (cell dynamics and tissue growth in engineered tissues) and industrial testing because it is non-destructive, label-free, high resolution, and quick [22–27]. As opposed to fluorescent or ionizing radiation, OCT depends on the scattering characteristics of the sample [28].

The field of medical imaging strategies has witnessed a transition from general tissue imaging to specialized vascular imaging. In the past, the focus primarily rested on capturing detailed anatomical structures and tissue composition. However, with the advent of advanced technologies, such as contrast-enhanced ultrasound, magnetic resonance angiography, and CT angiography, the emphasis has shifted towards non-invasive visualization of blood vessels and circulatory systems. This shift has allowed clinicians to study blood flow dynamics, and plan minimally invasive interventions with greater precision. For example, CT angiography combines a CT scan with an injection of a contrast agent and produces images of arteries in a body part [29]. Spiral CT scanners with 4–64 sections enables quick capture of isotropic data sets [30]. Utilizing the intrinsic nuclear spin characteristics of the nucleus to selectively detect and assess flow in a system of moving parts is a clinically beneficial application of MR angiography. Combining the flow experiment with surface coil techniques will result in high-resolution MR angiographic images [31, 32]. Typically, imaging vascularization is important for understanding healing processes of injured tissues and the relationship between blood vessels and host tissues, which can accelerate the development of treatment modalities. One of the advanced imaging techniques is the quantitative 3D imaging platform, which is a combinatory system of optical tissue clearing, light-sheet microscopy, and 3D image analysis [33]. While conventional techniques allow imaging of small regions (mm^2^ areas), the quantitative 3D imaging platform enables the capturing of 3D maps of skeletal progenitors and vessel subtypes in the whole murine calvarium at single-cell resolution. Also, the 3D maps can be quantitatively analyzed including platelet endothelial cell adhesion molecule-1, endomucin, and osterix expression, which suggests a detailed framework for vascularized craniofacial bone remodeling. In another study, hierarchical imaging was performed to attain 3D brain vascular networks using scanning electron microscopy, synchrotron radiation and desktop micro-CT imaging, and computational network analysis [34]. Network features were quantified in various aspects, such as directionality, vessel diameter, length, and tortuosity, branch point density, and vascular volume fraction, from the early postnatal to adult brain. The hierarchical imaging technique can provide a detailed morphological development of the entire murine brain vasculature, which can also be applied to biologically-relevant questions including angiogenesis, pathophysiology, and drug effects. In addition to above techniques, a hybrid *in-vivo* imaging technique, photoacoustic microscopy, was introduced and customized for a high signal-to-noise ratio and high resolution for microvascular imaging [35]. While many imaging techniques require contrast agents, photoacoustic microscopy successfully captured microvessels by localizing photoacoustic signals from red blood cells without using contrast agents. Then, vascular network in ears, eyes, and the brain of a mouse were reconstructed as a 3D photoacoustic map image, and the flow of red blood cells in the mouse ear was represented. Overall, vascular imaging techniques are rapidly developing in terms of high resolution and computational analysis, and the attained anatomical atlas will be a useful tool for confirming the reconstruction of vascularized tissue and organ substitutes.

Completion of 3D models requires designing of the internal architecture of tissue substitutes after the acquisition of 3D anatomy. The internal architecture includes vascular networks for the supply of nutrients, internal channels, and pores for proper cell attachments, proliferation, and migration. The most common approaches utilized in designing of internal structure entail computer-aided design (CAD), freeform design, image-based design, internal surfaces, etc [36]. The generated 3D model is then converted into stereolithography (STL) format, which is the most readable and accepted file format for most commercial bioprinters (figure 1(a)). Once a blueprint of 3D anatomical structures is obtained, selection of biomaterials should be followed according to tissue-specific mechanical and biological characteristics.

#### 2.1.1. Selection of biomaterials

Due to their similarity with tissue extracellular matrix (ECM), polymers, such as hydrogels, are widely used in tissue engineering. They can be of either naturally occurring or synthetically derived [37, 38]. Synthetic polymers have been found as the crucial elements in supporting cellular and biological activities throughout the bioprinting process. Synthetic polymers with controlled degradation properties, strong mechanical properties, tunable chemical structures and non-toxic degradation products have been considered for bioprinting of tissue and organ substitutes [39]. The synthetic polymers used for bioprinting are poly (lactic acid) (PLA), poly (glycolic acid) (PGA), poly(lactic-co-glycolic acid) (PLGA), polyurethane (PU), and polycaprolactone (PCL) [40–42]. These synthetic polymers are often utilized as physical frame structures for bioprinting, which improves handling of constructs from the bioprinting stage to *in vivo* implantation [9, 43]. Since hydrogels or bioprinted constructs can easily get deformed during their transfer post-bioprinting, improvement in handling can reduce the risk of deformation and provide a stable microenvironment for cells to proliferate and develop into tissues. In addition, synthetic polymers can generate synergistic effects with hydrogels like improving physical stability and biocompatibility when implanted for regeneration of hard tissues, such as bone and cartilage [44]. The most commonly and currently used natural polymers for fabrication of tissue and organ substitutes are alginate, collagen, gelatin, cellulose, hyaluronic acid, fibrinogen, agarose, and chitosan [40]. Methacrylated derivatives of gelatin, collagen, and hyaluronic acid have also been considered for various tissue biofabrication purposes [43, 45].

The selection of bioinks for bioprinting of tissue and organ substitutes depends on the characteristics, mechanical, structural, and biological properties of the target tissue and organ respectively. For example, to regenerate soft tissues like skin, natural polymers have been dominantly used over synthetic polymers because of their similarity to native ECM structure and composition promoting cell attachment, proliferation, and differentiation [46]. Especially, gelatin and collagen have been prevailing materials for skin bioprinting [47]. For fabricating hard tissue substitutes such as bone, scaffolds need to have similar properties to that of the human bone. Polymers such as chitosan, PLA, PLGA, PCL, salts of calcium and phosphate are used in bone bioprinting due to their inorganic and osteoconductive nature leading to bone growth [37]. The characteristics of natural and synthetic polymers with their applications in the bioprinting domain are presented in table 1. Despite the selection of tissue-specific bioinks is crucial for the success of engineered tissues, the use or incorporation of angiogenic biomaterials also plays an important role in prompting vascularization in these engineered tissues as described in the following section (figure 1(a)).

**Table 1.** An overview of the biomaterials relevant to 3D bioprinting including their characteristics and applications.

Polymers		Bioceramic	Bioprinting method	Advantages	Limitations	Applications	References
Natural	Synthetic						
Alginate			Inkjet, extrusion, droplet, light	Rapid gelation capability promoting high shape fidelity as a matrix, minimizes shear stress on cells	Low viability of endothelial cells, not supporting vascular morphogenesis	Sacrificial material for printing vascular structures, skin tissue engineering	[48–51]
Cellulose			Droplet, extrusion, light	Low cost, biocompatibility, sustainability, low cytotoxicity, antimicrobial properties, tunable degradation profile, used as thermoplastics	Limited cell adhesion, limited capability to support growth	Skin and wound dressing, bone tissue engineering, nerve tissue repair, ophthalmic tissue repair	[52–55]
Collagen type I			Droplet, extrusion, laser	Long term stability, ideal microenvironment for angiogenesis	Lack of sufficient mechanical properties, inferior printability	ECM for bioprinted vascular tissues, cartilage, liver, cornea	[48, 56–60]
Gelatin and Gelatin methacrylate (GelMA)			Extrusion, laser	High versatility, rapid crosslinking	Limited resolution in bioprinting (500–1000 *μ*m), require an extensive understanding to modulate mechanical properties	Liver, bone, cartilage, muscle tissue engineering	[44, 61–64]
Hyaluronic acid (HA)			Extrusion, light	Superior biocompatibility, capacity to create malleable hydrogels, shear thinning, sufficient viscosity	Lacks gelation ability	Skin, cartilage, bone, vessel tissue engineering	[65, 66]
Agarose			Extrusion, droplet	Self-gelling characteristics, water solubility, tunable mechanical properties, non-immunogenic characteristics	High stiffness may hinder cell spreading	Bone, vascular tissue engineering	[67–69]
Fibrinogen			Droplet, extrusion	Shear-thinning behavior, precise control over the amount of bioink and deposition rate	Possesses Newtonian behavior	Brain, cardiac, skin cartilage, bone tissues	[70–72]
Chitosan			Extrusion, laser	Antibacterial properties, porous structure of chitosan can modulate angiogenesis	Require acidic solution for dissolution	Fabrication of sponge scaffolds for wound dressings, cartilage regeneration, vascular tissue engineering	[73–76]
Decellularized extracellular matrix (dECM)			Extrusion	Retains the native tissue morphology including vasculature and biofactors	Slow gelation process, poor shape fidelity	Skeletal muscle tissue engineering	[77]
	Pluronic		Extrusion	Thermoreversible gelation behavior	Poor cell viability, weak mechanical properties, need a low temperature to liquefy (<4 °C)	Vascular tissue engineering	[78]
	Poly(lactic-co-glycolic acid) (PLGA)		Extrusion	Good mechanical properties, stability	Poor biocompatibility	Bone tissue	[78]
	Polyethylene glycol (PEG)		Extrusion, inkjet	Strong mechanical properties, non-immunogenic, non-cytotoxicity	Bioinert	Pancreatic tissue engineering, vascular, bone tissue engineering	[78, 79]
	Poly-vinyl alcohol (PVA)		Selective laser sintering (SLS) printing	Biocompatible, biodegradable, high tensile potency	Poor cell adhesion and proliferation	Cardiac tissue, articular cartilage	[80]
	Polylactic acid (PLA)		FDM	Biocompatibility, degradability,	Brittleness	Musculoskeletal tissue engineering	[78]
		Ti6Al4V	Laser beam melting	High strength, low density, nontoxic	Poor biocompatibility	Dental tissue engineering	[37]
		Calcium phosphate	Extrusion	Osteoconductive, good bioactivity, absorbability	Low compressive strength	Vascularized bone tissue	[37, 81]
		Biphasic calcium phosphate + Zirconia	Extrusion	Good mechanical and bone morphogenic properties	Limited zircornia concentration (<10 wt%) due to high viscosity hindering extrusion	Bone Tissue	[82]
Composites							
Natural polymers	Synthetic polymers	Bioceramics	Bioprinting method	Characteristics of the scaffold		Applications	References
Chitosan	PLLA		Fused deposition modeling (FDM)	• Good cell viability, better mechanical properties, anti-inflammatory • Higher degradation rate		Bone tissue scaffold	[83]
Chitosan	PCL		FDM	• Versatile to construct micro channel structure within scaffolds to promote angiogenesis		Vascularized bone tissue	[84]
GelMA + Alginate	Poly(ethylene glycol)-tetra-acrylate) (PEGTA)		Co-axial extrusion	• Precise high resolution printed constructs • Ultraviolet (UV) light-dependent gelation process of GelMA		Vascular tissue engineering	[85]
Hyaluronic	Polylactic acid (PLA)		Extrusion	• Improve cell functionality by an increase in the expression of chondrogenic gene markers		Articular cartilage	[86]
Alginate + Gelatin		58 S Bioactive glass	Extrusion	• No cytotoxicity, good bioactivity, improved porosity		Bone regeneration	[87]
Alginate	Polyethylene glycol diacrylate (PEGDA)	Calcium sulphate	Extrusion	• Complex cellularized structure with high viability of the cells.		Kidney	[88]
Collagen		HA	Extrusion	• Better biocompatibility, osteogenic differentiation • Difficult to exactly mimic the complex native tissue		Bone tissue	[89]
Phytagel	PVA		Extrusion	• Mimics the mechanical properties of human soft tissue and showed good cell attachment and viability		Soft connective tissue	[90]
Alginate	PVA	Hydroxyapatite	Extrusion	• Good printability, osteoconductive, biodegradable		Bone tissue	[91]
	PLGA	Hydroxyapatite	Laser stereolithography, extrusion	• Higher cellular growth, differentiation		Bone tissue	[92]

#### 2.1.2. Angiogenic biomaterials

Angiogenic biomaterials are substances designed to promote the formation of new blood vessels, a process crucial for various medical applications including tissue engineering and biofabrication. The fabrication of blood vessels capable of supplying essential nutrients and oxygen to cells located deep within the tissue and simultaneously removing waste is a key challenge in engineered tissues. The viability of cells around vessels in the engineered tissues is significantly influenced by their spatial distribution. Cells die for lack of oxygen and nutrients if they are more than 100–200 *µ*m from the closest blood vessel [93]. Therefore, this key point must be considered to develop angiogenic biomaterials and engineer the formation of the vasculature. Biomaterials must allow vascularization to attract cells, proliferate, and support the formation of tissue ECM. Considering the significance of the interaction between angiogenesis and tissue repair, it is crucial to develop biomaterials that boost angiogenic processes and eventually result in the production of re-vascularized tissue.

ECM components of biological origins are frequently used as biomaterials to promote angiogenesis and the creation of endothelial networks. During angiogenesis, cells engage in dynamic interactions with the ECM and are subsequently controlled by cues from the local microenvironment. Cells obtain signals for survival and growth from the ECM, which also acts as a mechanical scaffold, while orchestrating the precise coordination of both biochemical and biophysical cues in a complex spatiotemporal manner [94]. Under normal physiological conditions, dormant blood vessels are enveloped by a substantial basement membrane primarily composed of collagen type IV, fibronectin, and the adhesive protein laminin. During angiogenesis, cell-secreted proteases degrade this basement membrane, exposing budding endothelial cells to an interstitial ECM rich in type I collagen and elastin [95]. Cell migration and proliferation are encouraged by this environment [96]. The linkage between type I collagen and cell-surface integrins relies on adhesion glycoproteins present in the interstitial ECM, notably fibronectin and vitronectin, which play a pivotal role in the process of vascular development [97]. Incorporating sites for cell adhesion and protease sensitivity within the biomaterial is essential for promoting angiogenesis within the biomaterial or at the interface between the biomaterial and tissue [98]. Any alterations in the composition and structure of the ECM can influence cell behavior and angiogenesis by interacting with cell-surface integrins [94]. Also, understanding and recapitulating the important developmental stages of natural blood vessels is an attractive strategy to generate vasculature. In the course of human embryonic development, the first vascular structures originate through the spontaneous emergence of precursor cells, known as endothelial precursors, a phenomenon termed vasculogenesis. Conversely, angiogenesis describes the process wherein new blood vessels sprout from pre-existing ones. Under typical physiological circumstances, a diverse array of bioactive agents, including growth factors and cytokines, along with cell types like endothelial cells and signaling mediators like nitric oxide, work in concert to support the synchronized advancement of both vasculogenesis and angiogenesis [99].

A wide array of natural and synthetic biomaterials has been investigated for their ability to support angiogenesis. Materials including decellularized ECM (dECM), collagen, gelatin and fibrin mimic the blood vessel ECM, but they suffer from limited mechanical strength [100]. While biomaterials, such as alginate, dextran, hyaluronic acid and polyethylene glycol (PEG), have the required mechanical properties, they do not have the required bioactivity when utilized alone [98]. Bioinks incorporating dECM have gained attention because the decellularization process retains the native vessel microenvironment while eliminating cellular and nuclear components, thus fostering cell growth and creating non-immunogenic tissues. For example, Choi *et al* harnessed dECM to formulate a bioink, enabling granule-based coaxial bioprinting of the muscle tissue. Pre-vascularization of the resulting tissue using a vascular dECM-based bioink led to improved muscle function recovery and the prevention of hypoxia in a rat model of volumetric muscle loss [101]. In another study, a hybrid bioink comprising vascular-tissue-derived dECM (VdECM) and alginate was used to create bio-blood-vessels (BBVs) via bioprinting. These BBVs served as delivery system for endothelial progenitor cells (EPCs) and proangiogenic drugs to treat ischemic injuries. The approach enhanced EPC survival, differentiation, and neovascularization in a mouse hind limb ischemia model, leading to significant limb recovery. This study highlights the potential of the dECM-based bioink for treating ischemic diseases [102]. Furthermore, natural biomaterials such as collagen, fibrin, and gelatin have a simpler composition, making it easier to implement specific strategies for delivering angiogenic factors [103]. These biomaterials provide a conducive environment for cells due to their inherent cell-adhesive properties and sensitivity to proteases. Their biochemical and physical characteristics are primarily shaped by the innate ECM proteins and are not easily subject to extensive modification. In a study, researchers designed a layered 3D structure filled with neural stem cells. They bioprinted thrombin-crosslinked fibrin gel alongside collagen. Over 3 d, the fibrin gel functioned as a reservoir, gradually releasing vascular endothelial growth factor (VEGF). Notably, cells from the collagen structure migrated towards VEGF-releasing fibrin, which resulted in increased cell proliferation and the development of branched morphologies with neurite projections throughout the cell culture period. In contrast, control samples, where fibrin was bioprinted directly into collagen without VEGF or with VEGF, did not exhibit any signs of cell proliferation or migration [104]. Another polymer, gelatin methacryloyl (GelMA) has garnered substantial interest in tissue engineering due to its noncytotoxic and biodegradable nature. Its interest lies in its photocrosslinkable characteristics and customizable mechanical strength, all while preserving essential cell-binding motifs [105]. Using GelMA and fibrin, Calderon *et al* investigated the tubulogenic potential of induced pluripotent stem cells (iPSCs)-derived endothelial cells (iPSC-ECs) in these hydrogels, with and without supporting human mesenchymal stem cells (hMSCs). They used a dual-color lentiviral reporter to track key vascular morphogenesis steps, such as vacuole formation and lumen coalescence, in real time. The results confirmed that iPSC-ECs could form tubules in fibrin, and in GelMA, their tubulogenic response was enhanced when co-cultured with a small fraction of hMSCs. This work has implications for studying vasculogenesis and tissue engineering to pre-vascularize tissue constructs [106]. In another study, small-diameter blood vessels were fabricated comprising two distinct cell layers: vascular endothelial cells (VECs) and vascular smooth muscle cells (VSMCs). They formulated a bioink that incorporated VSMCs within GelMA/polyethylene(glycol)diacrylate/alginate along with lyase enzyme, aiming to replicate the natural vessel composition. Over time, both VSMCs in the scaffold and VECs in the lumen displayed significant proliferation. The authors reported these bioprinted blood vessels as promising candidates for small-diameter blood vessel replacements in clinical applications [107].

In a study conducted by Benning *et al*, an extensive comparison was performed among several commercially available bioinks. These bioinks were evaluated for their physicochemical properties, swelling/degradation behavior, influence on EC characteristics, and suitability for bioprinting. The primary objective was to identify the optimal bioink or combination thereof for inkjet bioprinting of ECs for creating prevascularized tissue constructs. While the majority of bioinks proved suitable for bioprinting, Pluronic F-127 and the alginate/gelatin blend exhibited rapid degradation. Agarose, Pluronic F-127, alginate, and alginate/gelatin were deemed unsuitable due to issues like non-adherence and an inability to support EC proliferation. In contrast, gelatin supported EC viability but did not facilitate sprouting, whereas fibrin and collagen emerged as the most suitable bioinks. These hydrogels demonstrated the capability to support vasculogenesis-related parameters and showed acceptable printability [108]. On the other hand, synthetic biomaterials, like PEG, display a distinct advantage due to their enhanced controllability when compared to natural biomaterials. These materials offer the ability to independently fine-tune various properties. To optimize for angiogenesis, these materials can be precisely adjusted for factors such as stiffness, crosslinking degree, the quantity, and specificity of cell-adhesion and protease-sensitive sites, as well as the integration of binding sites for angiogenic factors [109]. In addition to optimizing the inherent characteristics of biomaterials, including pore structure, surface chemistry, topographical features, and overall rigidity, a crucial strategy for promoting angiogenesis involves the precise delivery of angiogenic molecules and growth factors through these biomaterials.

Concurrently, nanomaterials are gaining attention in biomedical applications due to their unique properties in promoting angiogenesis [110]. They not only serve as efficient carriers for delivering crucial angiogenesis-related proteins and mRNA but also mimic nano-topological structures found in the primary ECM of blood vessels [111]. This unique property of nanomaterials stimulates the gene expression of angiogenic effects, facilitating and enhancing the process of angiogenesis. This multifaceted capability of nanomaterials holds great promise for promoting successful tissue regeneration and treating ischemic diseases by orchestrating the complex process of blood vessel formation. Additionally, nanomaterials, such as gold nanoparticles [112] or carbon nanotubes [113], can deliver angiogenic factors effectively and mimic the nanostructure of native blood vessel ECM, further promoting blood vessel growth. Hydrogels with bioactive components, like platelet-derived growth factor (PDGF)-loaded gelatin hydrogels, can enhance angiogenesis at wound sites [114]. Furthermore, graphene and its derivatives have shown promise in promoting angiogenesis. Graphene oxide can be functionalized with angiogenic growth factors and used as a scaffold to enhance blood vessel formation [115].

Overall, research on angiogenic biomaterials has made remarkable progress in recent years, offering promising avenues for medical applications such as regenerative medicine and disease treatment. Innovative biomaterials, including natural ECM-mimicking substances, synthetic polymers, and nanomaterials, have demonstrated their potential to enhance angiogenesis and support the development of functional vascular networks within engineered tissues. These biomaterials have the capacity to address critical challenges related to nutrient and oxygen supply in deep-seated tissues, thereby advancing tissue regeneration. Future research endeavors should aim to refine and customize bioink formulations for precise control over angiogenesis within tissue constructs. Leveraging advanced manufacturing techniques like 3D bioprinting will enable the creation of patient-specific vascularized tissue constructs, further enhancing their clinical relevance. Additionally, the exploration of stem cell-based therapies, particularly endothelial cells derived from iPSCs, holds promise for improving vascularization outcomes. Moreover, investigations into the long-term stability and functionality of vascular networks within engineered tissues, as well as their integration with the host circulatory system, remain critical areas of future work. Advancements in biomaterial design, combined with a deeper understanding of angiogenic processes, will continue to drive progress in tissue engineering and regenerative medicine, ultimately benefiting patients worldwide.

#### 2.1.3. Angiogenic factors in bioprinted vascular constructs

**Figure 2.**
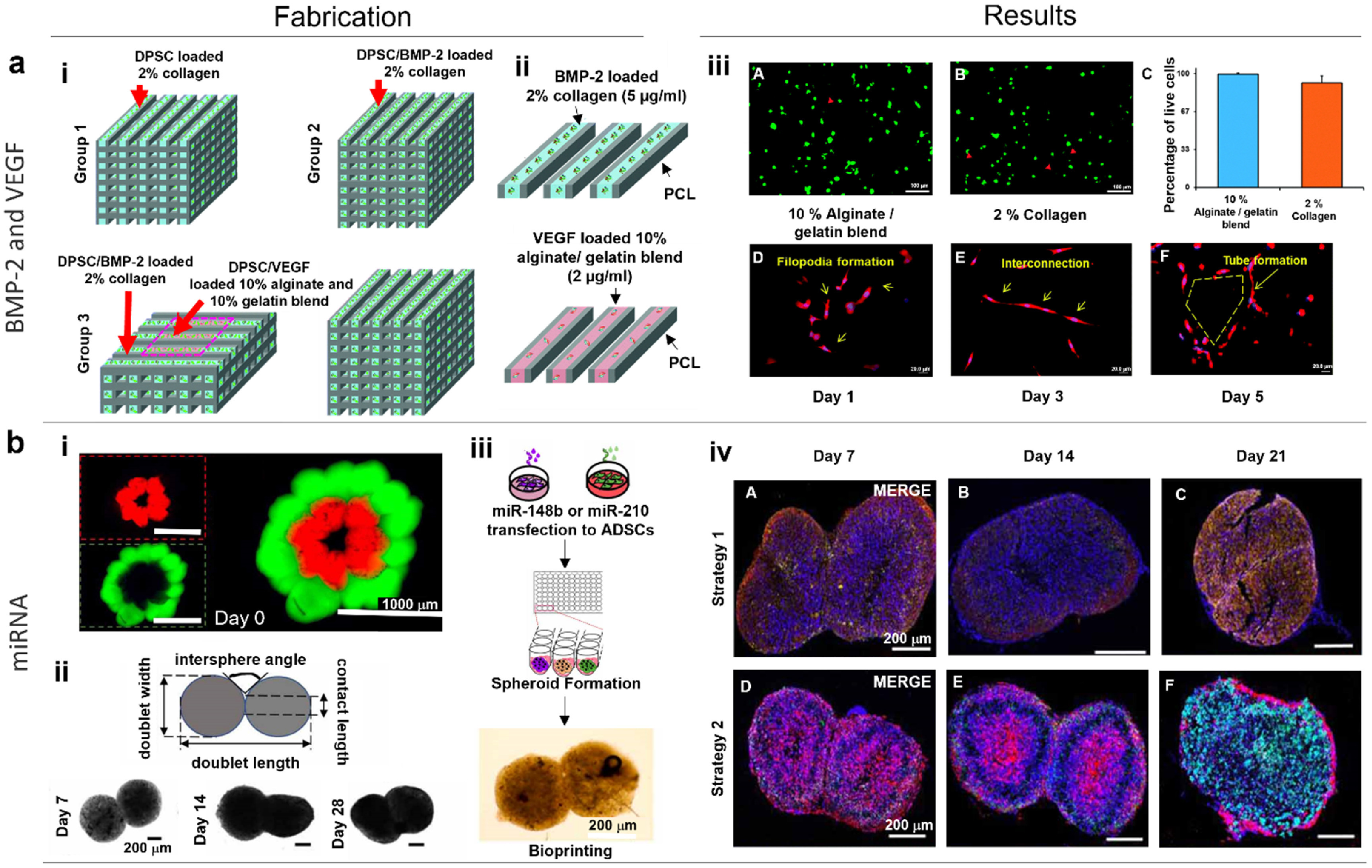
(a) 3D Bone regeneration with controlled BMP-2 and VEGF delivery. (i) Schematic representation of the three different structure designs: Group 1 shows a mesenchymal dental pulp-derived stem cell (DPSC) bioprinted structure utilizing 2% collagen, Group 2 shows a DPSC/BMP-2 bioprinted structure incorporating 2% collagen, and Group 3 shows a DPSC/dual growth factor bioprinted structure using a combination of 2% collagen and 10% alginate/10% gelatin blend. (ii) Schematic representation of the BMP-2 loaded collagen gel in a PCL frame and the VEGF loaded alginate/gelatin in a PCL frame. (iii) Confocal images showing DPSCs stained with calcein AM (green) and ethidium homodimer (red) for live and dead cells, respectively, on (A) 10% alginate/gelatin and (B) 2% collagen. (C) Quantification of live cell percentages. *Bandeiraea simplicifolia*-lectin staining of DPSCs in alginate/gelatin supplemented with VEGF at Days (D) 1, (E) 3, and (F) 5. Reproduced with permission from [123]. © The Royal Society of Chemistry 2015. CC BY-NC 3.0. (b) miRNA-enhanced pre-vascularized bone tissue. (i) Representative images of the Haversian canal model created using the aspiration-assisted bioprinting technique. Bioprinted structures comprising of ADSCs spheroids labeled with CellTracker™ green CMFDA dye and CellTracker™ Red CMTPX dye after bioprinting. (ii) Representative light microscopy images showing the fusion of spheroids within assembled doublet structures over a 21 day timeframe and the schematic representation of measured morphological parameters during the fusion process. (iii) Schematic representation of the biofabrication process for creating doublet structures using spheroids transfected with miR-210 and miR-148b. (iv) Immunostaining images depicting DAPI (blue), RUNX2 (green), and VE-cadherin (red) labeling in Strategy-1 doublets (2 d transfection period, followed by fabrication and culture of spheroids for 21 d) and Strategy-2 doublets (14 d transfection period, followed by fabrication and culture of spheroids for 21 d) at Days 7, 14, and 21. Reproduced from [124]. © IOP Publishing Ltd. All rights reserved.

As an angiogenic growth factor, VEGF is a key contributor in formation of new blood vessels; and endothelial cells respond favorably to VEGF, which triggers their proliferation and sprouting to produce new, immature artery sprouts [116]. Even though VEGF can induce angiogenesis, other elements are needed to encourage vessel maturation. For example, PDGF promotes development of the nascent arteries by stimulating and attracting pericytes and smooth muscle cells that interact with endothelial sprouts, maintaining them and limiting regression [117]. Other growth factors that directly or indirectly regulate angiogenesis include the acidic and basic fibroblast growth factors (FGF-1and FGF -2 [118], transforming growth factor-beta (TGF*β*) [119], angiogenin [120] and epidermal growth factor (EGF) [121]. For example, Meng *et al* developed 3D bioprinted metastatic model by replicating the structure of peripheral blood vessels and growth factors (such as EGF and VEGF) were released from stimuli-responsive capsules. Using 3D bioprinting, a molecular gradient of growth factors was created to replicate the biochemical properties of the tumor microenvironment, where lung cancer cells migrated towards arteries and invaded into the vascular system [122]. However, the model was relatively simple and a holistic, step-by-step integration of more constituents, such as immune cells, lymphatic vessels, etc., can be considered to mimic the heterogeneity of the tumor microenvironment. In another study, Park *et al* reported a method for pre-vascularizing bone tissue with dual growth factors. Human dental pulp stem cells that have both osteogenic and vasculogenic potential were bioprinted with bone morphogenetic protein-2 (BMP-2) in the peripheral zone of constructs and VEGF in the central zone, which was prone to hypoxia (figure 2(a)). Pre-vascularized constructs showed faster bone repair compared to non-vascularized tissues [123]. However, the constructs were tested in mouse dorsa only for 4 weeks and thus requiring their prolonged testing, if possible in larger animal models. Application of growth factors, in particular VEGF, has been the focus of efforts to stimulate blood vessel formation. However, it still raises concerns about the effect of released VEGF into the blood stream as there is a strong likelihood of undesired cell proliferation or tumorigenesis locally or distally.

In other works, endothelial cell-specific microRNA-126 (miR-126) have shown to promotes angiogenesis in response to angiogenic growth factors, such as VEGF or FGF, by suppressing opposing signal transduction pathway regulators [125]. It has also been shown that some stem cell populations can be inducted with endotheliogenic differentiation through miR-210, commonly known as the master hypoximiR (hypoxia-inducible miR) [126]. Using miRNA co-differentiation for 3D heterotypic pre-vascularized bone formation, Celik *et al* fabricated doublet structures using spheroids of ADSCs transfected with miR-148b and miR-210, and showed their osteogenic and endothelial differentiation, mineralization, and bone formation potential (figure 2(b)) [124]. Concurrently, as gene and DNA-based therapies continue to advance, there is an increasing need for plasmid DNA (pDNA) production and application. The use of pDNA to produce growth factors in transfected cells provides a powerful alternative and is beneficial in long-term effects with low costs as compared to purified protein. Chen *et al* formed mature blood vessels using electrospun fibers with loaded multiple pDNA-calcium phosphate nanoparticles. Fibers with encapsulated nanoparticles containing plasmids encoding VEGF (pVEGF) and basic FGF (pbFGF) led to significantly higher density of mature blood vessels compared to those containing individual plasmid [127]. However, the use of pDNAs poses a safety concern and needs more testing, which limits their clinical translation.

To induce vascularization in implanted tissues, the application of pro-angiogenic growth factors to recruit a host vasculature is a widely used strategy. However, the use of growth factors in large scale is a costly strategy and less effective *in vivo* due to their relatively slow release, which may also be unfavorable to cell viability especially immediately after implantation. Despite bioprinting of angiogenic factors is attractive in inducing angiogenesis, generation of perfusable macro-scale vascular constructs requires the use of bioprinting technologies in order to build such from scratch.

In addition to the bioactivity, angiogenic performance, and angiogenic factors of biomaterials, bioprinting modalities differ and add more criteria on selection of biomaterials, such as viscosity, print fidelity, and mechanical strength. Hence, biomaterials and bioprinting modalities are often required to be contemplated simultaneously; in this sense, the bioprinting stage including bioprinting modalities and related strategies on vascularization is elaborated.

### 2.2. Bioprinting stage

The bioprinting stage encompasses several different steps including the preparation of a bioink, bioprinter, and the bioprinting process. Bioprintability of the targeted tissue is influenced by several factors associated with the bioink and process. The bioink provide the process-ability for bioprinting to develop 3D constructs, where cells can proliferate and mature to form tissues or organs. The rheological properties of the bioink, such as viscosity, yield stress, and storage and loss modulus influence the print fidelity and the structural and mechanical strength of bioprinted constructs [18]. Although secondary cell lines have often been utilized, patient-specific primary or stem cells are ideal and suitable for the fabrication of transplantable tissue constructs to minimize rejection rates.

#### 2.2.1. Bioprinting modalities

For bioprinting, current process modalities include droplet-based bioprinting (DBB), laser-based bioprinting (LBB), extrusion-based bioprinting (EBB) and light-based techniques (figure 1(b)). DBB is a rapid technique, which ejects bioinks out of a nozzle on a substrate in form of droplets [128]. DBB is further classified into inkjet bioprinting (which generates droplets by thermal, piezoelectric and electrostatic technique), electro-hydrodynamic technique, acoustic technique and microvalve bioprinting [21]. DBB is a rapid, versatile technique; however, it has several drawbacks including clogging of nozzles and tubing line limiting the cell concentration to less than 10^6^ cells per ml, non-homogenous droplet size, and limited scalability of constructs [18]. LBB is one of bioprinting modalities that operates on the principle of laser energy for precise deposition and patterning of cells [18]. It is a nozzle-free process that allows minimal damage to cells and biological components. Nonetheless, it is often combined with other biofabrication techniques since it has poor scalability and limited stability. EBB utilizes pneumatic pressure or a mechanical force driven system to extrude bioinks [129]. Given its large deposition rate with continuous flow of bioinks, EBB is highly suitable for bioprinting scalable constructs. However, the employment of high pressure can be detrimental to cell viability [130]. As compared to other bioprinting techniques, EBB, on the other hand, enables faster bioprinting speed [131] with over 90% cell viability in general [132]. This strategy can also be combined with uniaxial or coaxial nozzles, which enable the generation of tissue construct with various structural configurations, such as hollow vascular constructs [133]. Light-based techniques involve the use of a concentrated ultraviolet (UV; *λ* = 320–380 nm) beam, which is irradiated onto a liquid photopolymer, and interpretation of a CAD model to generate the first layer [130]. Subsequent layers polymerize to form the desired solid structure. As UV light can be harmful to cells, blue light (*λ* = 350–400 nm) has been recently preferred as a light source for photo-crosslinking by fast polymerization kinetics and cell-friendly conditions [134]. Other wavelengths, such as green light (*λ* = 500–580 nm) and near-infrared light (*λ* = 900–1000 nm), have also been utilized to extend the applications of light-based techniques [135, 136]. STL is considered the oldest and the most mature light-based bioprinting techniques with high flexibility and resolution. These features make it useful for insulin delivery, corneal stromal tissue regeneration, and scaffold manufacturing [137]. Recently, newer techniques have evolved from STL, such as digital light processing (DLP) and volumetric bioprinting (VBP) [138]. DLP enables the curing of an entire layer directly. With its advantages, including high resolution and high-throughput, DLP has been applied with various biomaterials such as dECM, photo-crosslinkable hydrogels (i.e. GelMA), and ceramics (i.e. biphasic calcium phosphate) for liver, cartilage, and bone regeneration, respectively [139–141]. DLP also enables rapid integration of tissue constructs into organ-on-a-chip platforms for drug screening purposes [142]. While EBB allows building scalable tissue or organ substitutes with promising results, fabrication in a layer-by-layer fashion eventually leads to prolonged times. As one of novel bioprinting methods, VBP was introduced to build centimeter-scale constructs in ∼20 s using visible light projection on gelatin-based photo-responsive hydrogels via a spatially selective crosslinking method [143]. The biocompatibility of VBP was shown using articular chondroprogenitor cells and MSCs with high cell viability over 85%. The application of VBP can be expanded with various materials (including but not limited to alginate, HA, or dECM), stem cells, and organoids which can be utilized for developing heterogeneous structures. The characteristics of bioprinting processes are summarized in table 2 [144, 145].

**Table 2.** Characteristics of bioprinting processes with quantitative assessment and remarks.

Process		Resolution	Biocompatibility	Throughput	Precision	Scalability	Versatility	Cost efficiency	Remarks	References
Droplet-based bioprinting		⩾10 *μ*m	High	High	High	Low	High	Medium	- Rapid, versatile technique - Limited cell concentration (⩽10^6^ cells)	[18, 146, 147]
Laser-based bioprinting		⩾10 *μ*m	High	Medium	High	Low	Medium-low	Low	- Minimal damage to cells - Poor scalability and stability	[148]
Extrusion-based bioprinting		⩾100 *μ*m	Medium	High	Medium	High	High	High	- Capability of bioprinting scalable constructs - Low resolution	[131, 132, 149, 150]
Light-based bioprinting	Stereolithography	⩾50 *μ*m	Medium-high	Low	High	Medium	Medium	Medium	- Various therapeutic applications - Needs in improvement on scalability	[137, 150]
	Digital light processing	⩾10 *μ*m	High	High	High	Medium	Medium	Low	- High resolution and throughput - Curing precision may vary	[142, 151]
	Volumetric bioprinting	⩾100 *μ*m	High	Low	Medium	High	Medium	Low	- Rapid fabrication of centimeter-scale constructs - Needs in further verification on feasibility	[143]

Using various strategies, fabricating scalable tissue or organ substitutes via 3D bioprinting has been one of the ultimate goals in tissue engineering and its clinical applications. Developing scalable substitutes is necessary for successful clinical translation since such substitutes should have the capability to cover the entire defect size or match the size of tissues or organs to be restored. However, it has been challenging to provide cells with an environment favorable to guiding cellular activities and dimensions above the centimeter scale [143]. A bulky structure can easily cause necrosis of cells by hypoxic conditions and less chance of cell–cell interactions [143]. Moreover, stiff bioinks are more compatible for stable structures and shape fidelity, but often hinder cell spreading, proliferation, or differentiation [152]. In contrast, bioinks suitable with low stiffness often lack mechanical properties but facilitate cellular activities. Despite these concerns, some bioprinting modalities (i.e. EBB and light-based techniques) have shown capacity toward the construction of volumetric structures. Further, a bioprinting method should not only have the capability of developing scalable structures but also consider resolution, printability, speed, biocompatibility, cost, and other relative parameters. In this regard, an in-depth multi-disciplinary investigation should be carried out for attaining scalable tissue and organ substitutes. For instance, the integration of 3D bioprinting and in vivo procedures was proposed to fabricate in vivo-engineered ECM matrix scaffolds [153]. Here, a PCL frame was used as a sacrificial template for cellularization when implanted subcutaneously in Sprague–Dawley rats. After 4 weeks, the cellularized templates were harvested, and the PCL frame and cells were removed to obtain ECM scaffolds. Because of the presence of abundant bioactive molecules, ECM scaffolds enhanced vascularization and cell infiltration upon in vivo implantation. These novel techniques have shown the possibility to develop volumetric tissue or organ substitutes by providing flexibility and versatility.

Nonetheless, tissue survival post-implantation heavily depends on sufficient nutrition and oxygen exchange via vasculature. As vascularization is a key factor for developing tissue and organ substitutes [154], the below section describes direct and indirect bioprinting approaches using aforementioned modalities for fabrication of vascular constructs.

#### 2.2.2. Bioprinting approaches for vascularization strategies

##### 2.2.2.1. Direct bioprinting for vascular tissue fabrication

**Figure 3.**
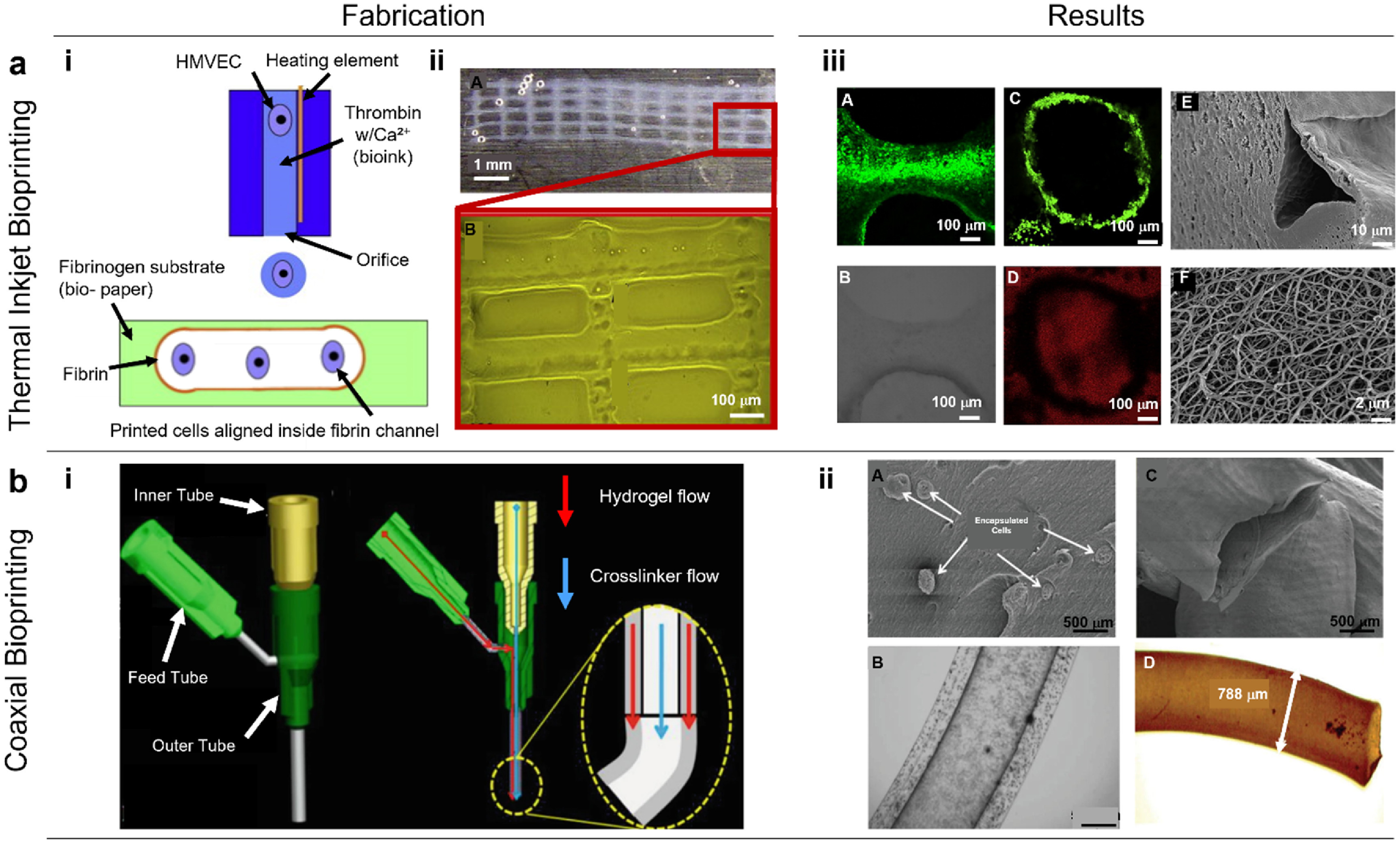
(a) Thermal inkjet bioprinting of microvasculature. (i) A schematic representation illustrating the deposition of human microvascular endothelial cells (HMVECs) in thrombin bioink using a modified thermal inkjet printer. The bioink containing cells was bioprinted into a fibrinogen substrate, allowing for the formation of fibrin channels while simultaneously depositing the cells into the scaffold. Bioprinted cells aligned within the fibrin channels and were poised for proliferation. (ii) A fibrin scaffold fabricated using a modified thermal inkjet printer which maintained its shape and structure post bioprinting. (iii) Channel structure of the bioprinted microvasculature cultured for 21 d. (A) Fluorescent staining (LIVE/DEAD) revealed bioprinted cells aligned within the fibrin scaffold. (B) Differential interference contrast (DIC) image showing the fibrin scaffold. (C) Bioprinted ring-shaped microvasculature after 21 d of culture. (D) When Texas Red-conjugated Dextran molecules were applied to the bioprinted structure, its integrated channel structure was able to expel dextran molecules via proliferated endothelial cells, which formed a robust and functional barrier. SEM images of bioprinted fibrin fiber: (E) cross-sectional view of a critically-dried fibrin fiber, revealing the channel structure at the cut section. The hole indicates the hollow nature of fibers for cell seeding and proliferation. (F) Close-up view highlighting the presence of nano-sized fibers on the surface of the bioprinted fibrin scaffold, facilitating cell attachment and proliferation. Reprinted from [156], Copyright (2009), with permission from Elsevier. (b) 3D coaxial bioprinting of vascular constructs. (i) 3D model representation of the coaxial nozzle and cross-sectional view of the coaxial nozzle assembly model illustrating the fluid flow paths for alginate and crosslinker solutions. Reproduced from [157], with permission from Springer Nature. (ii) (A) SEM image demonstrating cells encapsulated within bioprinted constructs. (B) A light microscopic image illustrating cell encapsulation and clear identification of the lumen in the center. (C) An SEM and (D) optical microscope image showing dehydrated 5% alginate constructs. Reproduced from [158] with permission from the Royal Society of Chemistry.

The direct bioprinting approach utilizes cell-encapsulated bioinks to actively bioprint hollow vascular constructs. Thus, researchers preferred blending different materials to optimize a suitable balance between mechanical strength and bioactivity for bioprinting. For example, Collins and Birkinshaw developed a bioink comprising of GelMA, sodium alginate, and 4-arm poly(ethylene glycol)-tetra-acrylate (PEGTA) to 3D bioprint perfusable vascular structures with a multilayered coaxial extrusion system [85]. However, the compressive moduli of the constructs significantly decreased after 21 d of culture, mainly due to the degradation of the GelMA component. Thus, it was challenging to test some of essential mechanical parameters, such as suture retention and burst pressure, and to keep the perfusability of constructs beyond 21 d. The bioink supported the growth and proliferation of encapsulated endothelial and stem cells, resulting in the development of viable vasculature [85]. Hinton *et al* reported a customized EBB technique called, freeform reversible embedding of suspended hydrogels (FRESH), for precise deposition of bioinks into a secondary support bath to maintain their structural integrity. The study demonstrated the ability to fabricate a part of the right coronary artery vascular tree with a hollow lumen with a wall thickness of <1 mm [155]. FRESH bioprinting showed capabilities to engineer soft hydrogels into complex structures; however, the direct bioprinting of functional tissues requires further research and development to become fully realized. In another study, Cui and Boland used DBB (inkjet bioprinting) to precisely manufacture micron-sized fibrin tubes. Human microvascular endothelial cells and fibrin were bioprinted together where the cells eventually arranged themselves and proliferated inside the channels (figure 3(a)) [156]. Using LBB, Wu *et al* printed simple branch/stem structures of human umbilical vein endothelial cells (HUVECs) and human umbilical vein smooth muscle cells (HUVSMCs) onto a thin hydrogel substrate. The structure mimicked the vascular networks in native tissue and allowed cells to develop new, finer structures away from the stem and branches. When HUVSMCs and HUVECs were bioprinted near one another, they exhibited a symbiotic interaction and form cell–cell junctions around lumen-like structures [156]. However, these were preliminary results in the creation of a self-assembling, artificially-guided vascular network, which requires comprehending the connection between HUVSMCs and HUVECs.

The use of coaxial nozzles in EBB revolutionized the fabrication of vascular constructs and resulted in the ability to bioprint lumen-incorporated strands. It involves a configuration of concentric nozzles wherein a bioink within the core is encapsulated by a shell crosslinker and on reversing this configuration allows for the deposition of hollow fibers. The diameter of the nozzle often requires optimization wherein the resolution could be improved upon decreasing diameter. The second factor is the flow rates of the shell and the core, which could be tuned to produce fibers of different configuration and shapes. In this regard, Zhang *et al* used coaxial printhead for direct hollow fiber fabrication, eliminating the need for any post-processing steps that are vital in other methods. The bioprinted hollow fibers were composed of either cell-laden chitosan or alginate hydrogels with a lumen diameter of less than 200 *μ*m [159]. In another study, Zhang *et al* encapsulated HUVSMCs in sodium alginate and bioprinted them in the form of vascular conduits using a coaxial nozzle, where cells exhibited growth with deposition of collagen and smooth muscle matrix on and around the lumenal surface (figure 3(b)) [158]. Hong *et al* reported coaxial bioprinting of vascularized tissues using a human dermal fibroblast (HDF)-laden gelatin-PEG-tyramine (GPT) prepolymer-based bioink in the shell and gelatin-loaded with HUVECs in the core. Tyramine and gelatin were separated by PEG in the GPT process, resulting in a quick gelation time of about 4.5 s. The perfusable vascular constructs were maintained for up to 8 days *in vitro* [160]. Nevertheless, the study requires further understanding on controlling the spatial distribution of bioprinted cells during long-term tissue maturation. In another study, Shao *et al* employed coaxial bioprinting with HUVECs-laden GelMA bioink in the core surrounded by alginate in the shell resulting in formation of vessel-like structures [161]. Yet, these endothelialized microfibers were not hollow during culture, which requires further investigation. In another study, Gao *et al* created a hybrid bioink composed of VdECM and alginate mixed with EPCs and proangiogenic drugs/PLGA microspheres, which promoted EPC differentiation, proliferation, and neovascularization allowing direct fabrication of blood-vessel structures [102]. However, low mechanical strength of vessels was a weakness of the study hampering its surgical anastomosis with the host blood vessel in the implantation process. Recently, Yu *et al* adopted a modified coaxial nozzle with two core needles to extrude double-channel gel fibers to simultaneously build perfusable multi-material constructs [162]. The filament in one channel can play a core/shell role, while the filament in the other channel can play a perfusion role. These parallel channels within filaments were separated by a ∼50 *μ*m wall of alginate, which is advantageous for nutrient supplementation via perfusion. However, the customized dual-core coaxial nozzle has not been tested for vascular applications. In another approach, small-diameter blood vessel grafts containing both functional endothelial and muscular cell layers were fabricated. For this, direct reservoir-assisted triple-coaxial cell printing and ECM bioinks obtained from the vascular tissue were used. The prematurely developed vessel was implanted in rat abdominal aorta for 3 weeks, which demonstrated patency, re-endothelialization, modified smooth muscle, and engraftment with the host tissue [163]. Although this approach accurately recapitulates small blood vessels (i.e., arteriole), it may not be suitable to study larger vessels (i.e. arteries, aorta). In another study, Gold *et al* developed a nanoengineered ECM bioink, composed of GelMA, poly(ethylene glycol) diacrylate (PEGDA), and 2D nanosilicates. The bioink was bioprinted into 3D cylindrical constructs comprising co-culture of vascular smooth muscle cells and endothelial cells. The 3D bioprinted constructs recapitulated thromboinflammatory reactions observed in advanced preclinical models or *in vivo* upon cytokine stimulation [164]. Of late, strategies leveraging the advantages of both microfluidics and coaxial printheads have also been reported [165]. Using this approach, it is possible to bioprint with low viscosity bioinks and still get a higher level of histoarchitectural complexity by mounting the chip upstream of a coaxial dispenser. Additionally, this can simultaneously deliver bioinks and crosslinking agents as separate flow streams through the coaxial nozzle, which allows single-step generation of stand-alone, hollow vascular conduits [166]. For example, Pi *et al* developed a multichannel coaxial extrusion system for microfluidic bioprinting of circumferentially multilayered tubular tissues in a single step, using customized bioinks consisting of GelMA, alginate, and eight-arm poly(ethylene glycol) acrylate with a tripentaerythritol core [167]. Recently, Ching *et al* developed a microfluidics-enabled molding approach combined with coaxial bioprinting to fabricate cell-laden vascular models. Freestanding and perfusable vascular structures with complicated shapes were fabricated using 3D porous molds of poly(ethylene glycol) diacrylate as casting templates that gradually release calcium ions as a crosslinking agent. The fabricated vasculatures could recapitulate physiologically-relevant conditions related to vascular diseases [168].

Direct bioprinting of vascular constructs has resulted in some successful *in-vivo* outcomes [169] but factors including immunological response, adverse host response, appropriate degradation rate, fibrosis, and necrosis due to limited nutrient diffusion and mechanical compatibility with the native tissue limit their wide applications [170]. This has motivated researchers to develop scaffold-free strategies. The first seminal study towards this was accomplished by Norotte *et al*, who used vascular cells including smooth muscle cells and fibroblasts, which were assembled into distinct units that were either multicellular spheroids or cylinders with regulated diameters (300–500 *µ*m). These were bioprinted layer-by-layer alongside with agarose rods, used here as a molding template. The individual components were combined after bioprinting to create single- and double-layered small-diameter vascular tubes (outer diameter ranging from 0.9 to 2.5 mm) with distinct shapes and branched structures [171]. However, limitations of the study include cell apoptosis due to thick vascular wall, limited resolution, and the inability to form complex geometries. In another study, Tan *et al* used 3D printed alginate hydrogel molds to facilitate spheroid fusion processes. Ring-shaped alginate molds were fabricated to obtain toroid-shaped tissue units where tissue spheroids, consisting of endothelial and smooth muscle cells, were then robotically deposited into the molds, which underwent fusion to form vascular constructs. However, additional calibrations and system enhancements will be required for the creation of non-open-structured molds to generate vasculature at smaller diameters [172]. Despite these intense efforts to bioprint vascular constructs, factors such as sufficient mechanical strength for structural integrity, requiring greater time to mature before enough ECM can be deposited, and scaling up structures to produce larger tissues still pose significant challenges. As an alternative to direct bioprinting, indirect bioprinting has a higher degree of freedom to build intricate structures and capability to encapsulate biomaterials within perfusable channels. Hence, detailed principles and applications of indirect bioprinting are followed.

##### 2.2.2.2. Indirect bioprinting for vascularized tissue fabrication

**Figure 4.**
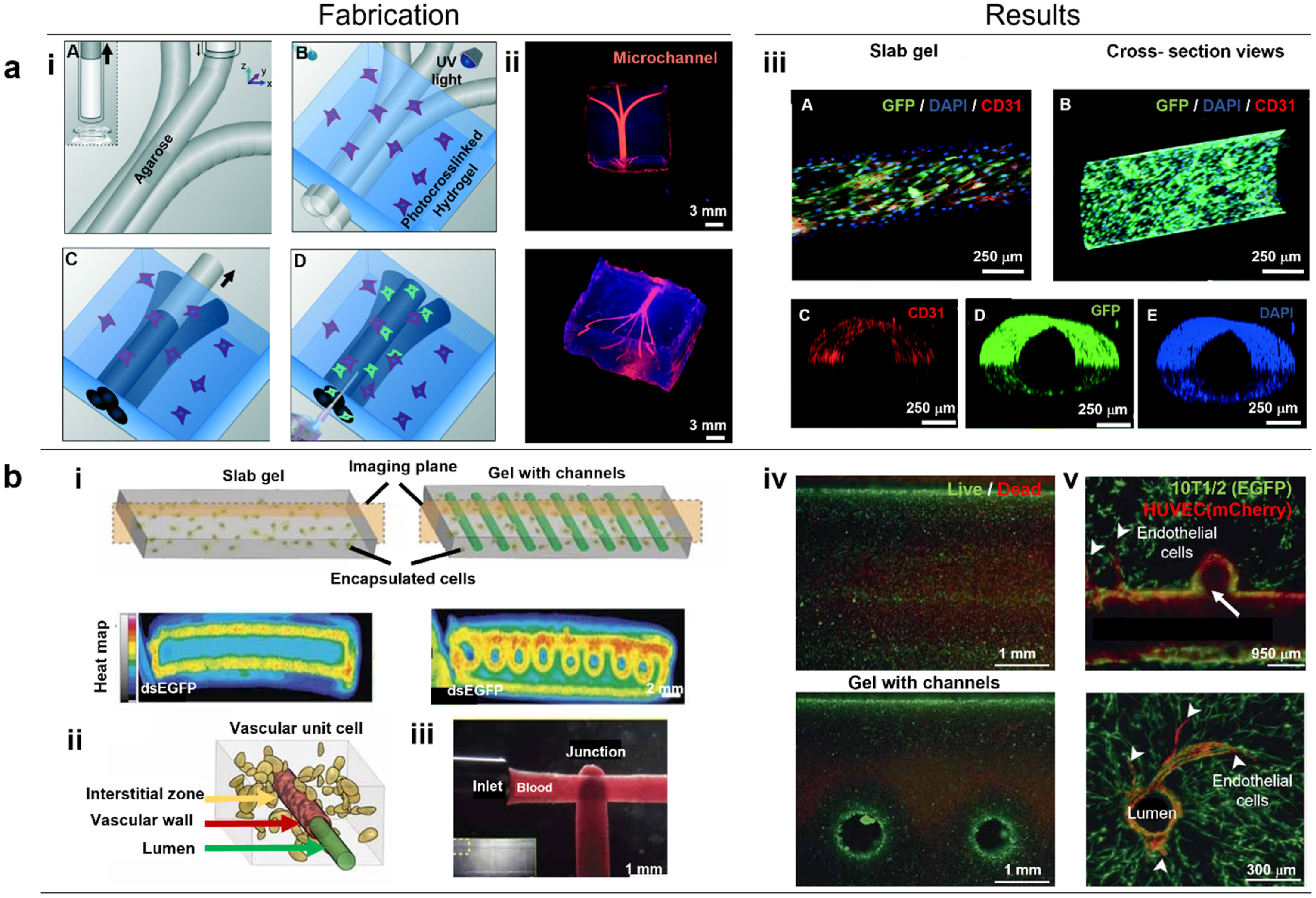
(a) Bioprinting vascular networks using sacrificial inks. (i) Schematic representation of the bioprinting process for agarose template fibers and micromolding for the channel formation. (A) A bioprinter equipped with a piston inside a glass capillary aspirated agarose. After gelation at 4 °C, agarose fibers were bioprinted at predetermined locations. (B) A hydrogel precursor was poured over the bioprinted mold and subsequently photocrosslinked. (C) The template was removed from the photocrosslinked gel that surrounds it, (D) which allows perfusion. (ii) The respective network was observed after perfusion, showing a 3D branching network obtained post-perfusion. (iii) Representative confocal and fluorescent microscopy images showing the immunostained HUVECs forming a monolayer within microchannels of various diameters after 7 d. (A) A confocal image displaying GFP/DAPI/CD31 markers indicating the HUVEC monolayer inside a 500 *μ*m channel. (B) Longitudinal view of *z*-stacked confocal images illustrating the HUVEC-lined microchannel, with the inset showing a cross-section view of the channel. Additional images of (C) CD31, (D) GFP, and (E) DAPI markers demonstrating the complete lining of the channel lumen. Reproduced from [173] with permission from the Royal Society of Chemistry. (b) Perfusable vascular networks. (i) Representative cross-section images illustrating PEG hydrogel with 40 × 10^6^ human embryonic kidney cells/ml after 3 days in culture. The intracellular double-stranded enhanced green fluorescent protein reporter demonstrated cellular activity in the outer region of the gel slab and in a circular pattern surrounding the perfusion channels. (ii) Schematic representation of the three compartments within a ‘vascular unit cell’ including the vascular lumen, endothelial cells forming the vascular wall, and the interstitial zone comprising the matrix and encapsulated cells. (iii) Patterned vascular channels enabling positive pressure and pulsatile flow of human blood, with intervessel junctions facilitating branched fluid flow. (iv) Fluorescent LIVE/DEAD assay staining (green, Calcein AM; red, Ethidium Homodimer) of primary rat hepatocytes and stabilizing stromal fibroblasts in agarose gels (slab versus channeled) after 8 days of culture. Cell survival was prominent at the gel perimeter and near perfused channels, while survival decreased progressively deeper into the gels. (v) Within the patterned vasculature, endothelial cells exhibited the formation of single and multicellular sprouts (indicated by arrowheads). Subsequent imaging at a deeper level (above image) provided confirmation of the continuous openness of the vascular lumen across vessels and intervessel junctions, as well as the sprouting of endothelial cells from larger vessels (arrowheads). Reproduced from [174], with permission from Springer Nature.

Indirect bioprinting utilizes a sacrificial ink, which is first deposited in a hydrogel matrix and then removed to create structures that resemble hollow vessels. These vessels can then be populated with endothelial as well as smooth muscle cells. Towards an effective technique for vascularization of tissue constructs, Bertassoni *et al* reported a method using 3D printed agarose template fibers, which were later removed for creating perfusable networks inside GelMA (figure 4(a)) [173]. Further, using a casting approach, Miller *et al* 3D printed rigid filament networks of carbohydrate glass comprising of a mixture of glucose, sucrose, and dextran as a fugitive phase. Several hydrogels, including PEGDA, fibrin-fibrinogen-thrombin, and alginate were investigated to form different vascular architectures with viable endothelial cells inside perfusable channels (figure 4(b)) [174]. For example, using DBB, Lee *et al* fabricated perfusable functional vascular channels using bioprinting of collagen blocks and HUVEC-gelatin mixture, where gelatin was eventually liquified to obtain tubular channels. Confluent endothelium was formed inside the bioprinted constructs, and it was demonstrated that under physiological conditions, cells up to 5 mm away from the channel could still survive [175]. In another study, Kolesky *et al* layer-by-layer co-bioprinted human neonatal dermal fibroblast-loaded GelMA strands, fugitive ink, and fibroblast-laden GelMA. Fabricated vascular network was enclosed in GelMA, and the fugitive ink (Pluronic F-127) was eliminated by liquifying it at 4 °C. More than 95% of HUVECs were found to be viable with a confluent EC layer formed 48 h after seeding [176].

Although the indirect bioprinting approach for vascularization of tissue constructs has advanced significantly due to the ease with building thick or slab gels as opposed to free-standing vascular constructs, such thick gel constructs may exhibit challenges including the inability to recapitulate multiple layers of blood vessels, inappropriate degradation of the bulk gel, and due to the intricacies involved with sacrificial inks, the size, morphology, and functionality of the obtained vessel-like structures may be constrained. Although indirect approaches are highly appealing, for fabrication of organ-on-chip platforms, their utilization in implantable tissues is limited as they usually lack suturable vascular pedicles to anastomose to the host vessel system.

### 2.3. Post-bioprinting stage

After bioprinting tissue and organ substitutes, the stage of post-bioprinting plays a critical role to differentiate stem cells and mature bioprinted substitutes in suitable bioreactors, which is a highly time-dependent process and performed under tightly controlled conditions (figure 1(c)). Cell–cell interactions, cellular differentiation and proliferation, and degradation of bioinks are controlled and coordinated during the post-processing stage to mimic the natural biological environment. Various stimulating factors are regulated inside bioreactors to modulate and enhance the maturation and vascularization of bioprinted tissue and organ substitutes. During this stage, sacrificial materials (such as vascular templates) can be removed either immediately or in controlled programmed manner to generate vascular networks [177]. This stage also includes the removal of the support material, if only, incorporated at the bioprinting stage to stabilize the constructs [18]. After vascular networks are generated, several strategies can be considered for maturation of blood vessels using bioreactors such as perfusion, rotational, or pressure bioreactors. To elaborate, perfusion bioreactors are composed of media, sensors, pumps, grafts, and chambers [178]. They enable dynamic stimulation on vasculature with controlled pressure, flow, and shear stress, mimicking physiological forces. For instance, a study proposed perfusable tubular scaffolds (20 mm in diameter and 2 mm in thickness) grown in a customized perfusion bioreactor [179]. The luminal surface of scaffolds was coated with fibronectin for functionalization purposes. When controlled flow rate was applied, the scaffolds revealed fluid flow without leakage and accelerated endothelial cell proliferation. Although perfusion bioreactors have proven to effectively mimic physiological stimulation and induce various cellular activities, their complex setup may hinder broader accessibility and clinical applicability. Rotational bioreactors can be utilized with a relatively simpler setup, which can be employed for cell seeding and development of cell-loaded tubular sheet structures [180, 181]. Also, smooth-muscle cells have been shown to elongate toward the flow direction, so bilayer blood vessel-like constructs can be achieved with control of rotating directions and use of multiple cell types (i.e., endothelial, smooth muscle, and fibroblast cells). However, the developed construct should be eventually tested with fluid flow as carotid arteries experience dynamic conditions with 60–120 mmHg pressure, 15–43 cm s^−1^ flow velocity, and 6–21 dyne cm^−2^ wall shear stress [182, 183], in which hemodynamic conditions can be recapitulated with pressure bioreactors *in vitro*. For the proof of this concept, several customized or commercially-available pressure bioreactors have been reported with different levels of complexity [184–188]. As compared to static conditions, smooth muscle organization and endothelial coverage were preserved in the vessel wall under pulsating conditions created by a pressure bioreactor [189]. Although bioreactors often require re-intervention and have not yet met off-the-shelf simplicity, the development of bioreactors will suggest a promising way to culture viable vessels and a patient-relevant system.

## 3. Advances in other vascularization strategies that can be coupled with bioprinted tissue and organ substitutes

Incorporation of complex hierarchal vascular network into bioprinted tissue constructs is highly significant for oxygenation; however, inclusion of multi-scale networks spanning from arteries and veins down to capillaries still remain the main challenge for fabricating scalable grafts. In this regard, other strategies can be coupled with bioprinting strategies to build vascularized tissues with multi-scale blood vessel networks.

**Figure 5.**
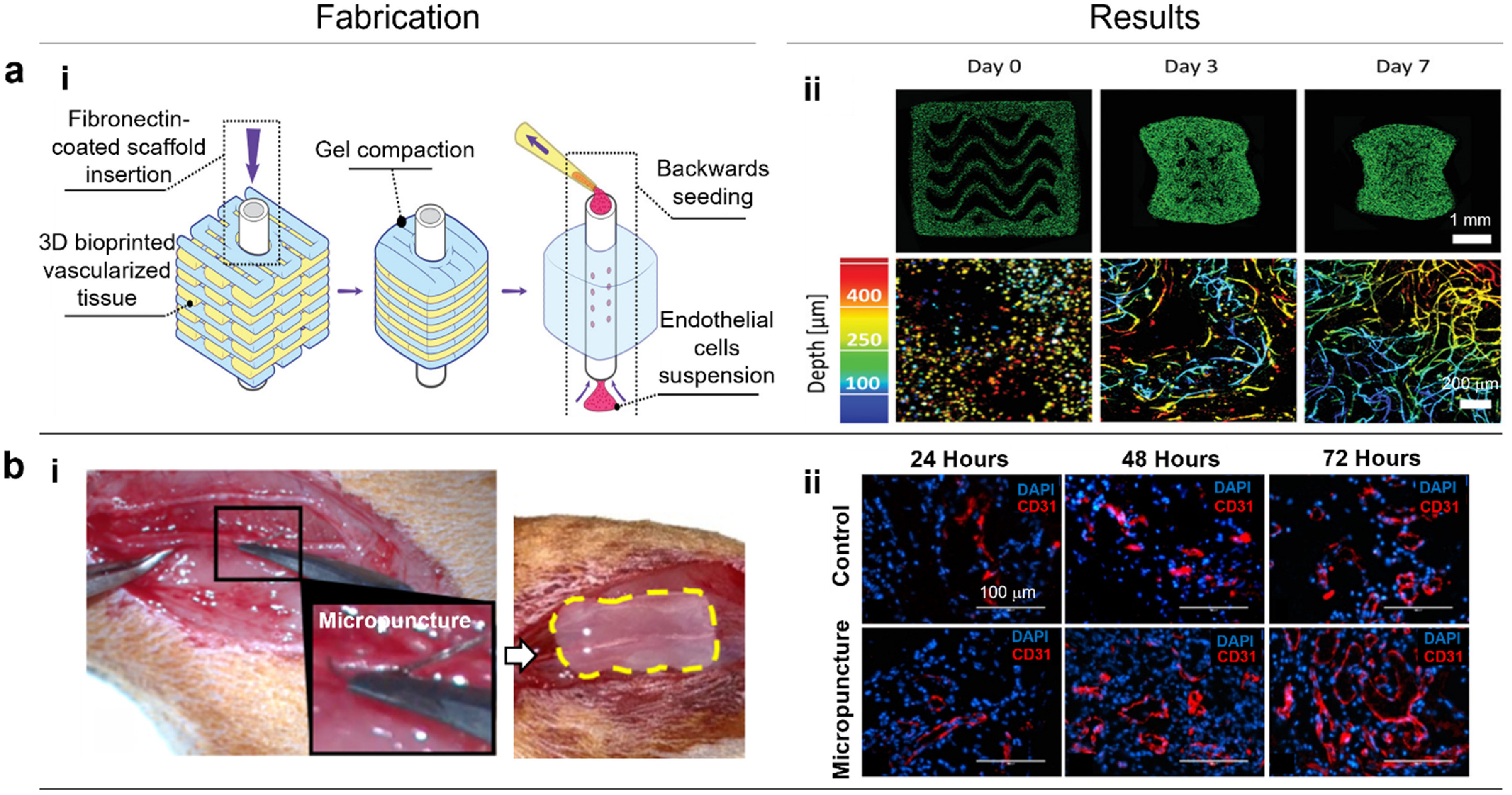
(a) Vascularized bioprinted tissue flaps. (i) Following 3D printing, a fibronectin-coated VascFold was promptly inserted into the tissue channel. The construct was cultured for 2 days, allowing cells within rhCollMA to undergo organization and form functional tissues. As the cells exerted forces, the gel underwent compaction, providing stabilization around the scaffold and concealing its side fenestrations. Subsequently, endothelial cells were seeded into the lumenal side by applying negative pressure. (ii) Confocal images depict the progressive formation of a vessel network within 3D bioprinted constructs at Days 0, 3, and 7. The top row illustrates the entire construct, highlighting the organization and vessel formation of ZsGreen-labeled endothelial cells (ZsGreen-ECs). The bottom row displays higher magnification confocal images, utilizing a depth color-coded projection to visualize the engineered vessel networks in detail. [12] John Wiley & Sons. © 2021 The Authors. Advanced Materials published by Wiley-VCH GmbH. (b) Micropuncture for accelerating angiogenesis. (i) The surgical approach involved creating segmental transmural micropunctures in the rat femoral artery and vein, followed by the direct placement of a collagen scaffold over the recipient vessels. The collagen scaffold was highlighted with a dashed yellow line, outlining its precise positioning in relation to the vessels. (ii) The collagen scaffolds on the micropunctured arteries were captured at 24, 48 and 72 h (DAPI in blue and CD31 in red). Micropunctured scaffolds exhibited accelerated and improved formation of luminal endothelial lining at all time points when compared to the non-micropunctured control. Reprinted from [190], Copyright (2021), with permission from Elsevier.

Hybrid strategies or strategies involving novel surgical methods have been introduced recently. For example, to achieve the combined mesoscale and microscale vascular networks, self-assembling microvasculature was bioprinted on ECM, which was then connected to the interior of a larger implantable tubular vascular scaffold (figure 5(a)) [12]. This resulted in the generation of engineered tissue flaps, where the microvessels obtained nutrients from the underlying tubular vascular scaffold. Sacrificial molds were also used to create the tubular vascular scaffold. The resulting constructs were accommodated in the lumen of a 3D printed construct resembling the alternating layers of recombinant human collagen methacrylate (rhCollMA) containing ECs, human adipose microvascular endothelial cells, support cells (SCs), and dental pulp stem cells, in one layer, and tissue-specific cells in another layer. ECs and SCs spontaneously formed the microvascular network. Later ECs were embedded in the inner lumen of the tubular vascular scaffold, creating the endothelium-like structure. This induced the formation of a hierarchal vascular network. Finally, utilizing a microsurgical technique, engineered constructs were then implanted into Spargue–Dawley rats by directly anastomosing to the femoral artery. Post-implantation, contrast microCT imaging and lectin perfusion of the constructs verified the vascular ingrowth.

Recently, a novel microsurgical technique enabling the creation of vascularized and thicker and clinically-translatable tissue constructs was demonstrated as shown in figure 5(b) [190]. The factors responsible for the promotion of neovessel formation in the neighboring scaffold was studied by the creation of segmental 60 *µ*m micropunctures in rat femoral arteries and veins, followed by injection of collagen. Detailed analysis of the harvested scaffolds at 24, 48, 72, and 96 h post-implantation revealed vigorous infiltration of ECs, macrophages, vascular endothelial growth factor receptor 2 (VEGFR2), increased physiologic perfusion, and rapid formation of capillary networks and thereby attributing to the overall increase in the quantity of small vessels. Despite this surgical technique being utilized in absence of bioprinting, it can be also combined with bioprinting for accelerating vascularization in bioprinted scalable tissue and organ substitutes.

## 4. Examples of bioprinted vascularized tissues

Although advances have been made in vascularization strategies for bioprinted tissue and organ substitutes to create long-term viable constructs for transplantation, incorporation of fully functional vascular networks is yet a milestone in the realm of tissue engineering and regenerative medicine. Despite such a milestone has not been reached yet, various vascularized tissues spanning from musculoskeletal tissues to tissues pertaining solid organs have been demonstrated as below (figure 1(c)).

### 4.1. Vascularized skin

**Figure 6.**
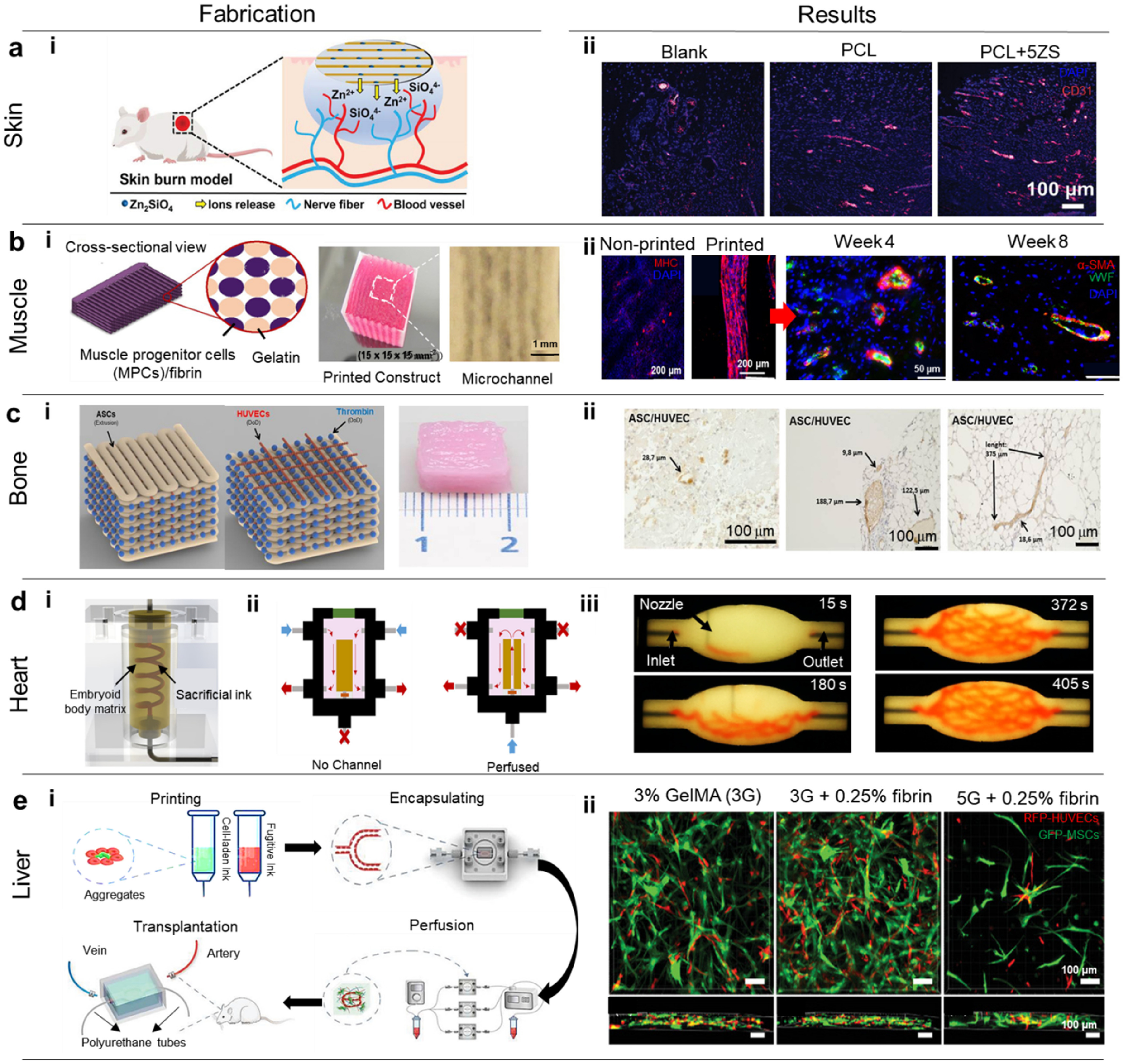
Bioprinting of vascularized tissues. (a) Vascularized skin repair. (i) A schematic representation depicts the fabrication of nanofibrous scaffolds composed of zinc silicate (ZS) for healing innervated and vascularized skin burn wounds. The multifunctional scaffolds, incorporating ZS nanoparticles, exhibit dual capabilities in promoting skin burn wound healing and facilitating neurovascular network regeneration. (ii) Evaluation of blood vessel in the wound region after various treatment (PCL and PCL + 5ZS) with respect to CD31 (red) on the 8th day. [195] John Wiley & Sons. © 2022 Wiley-VCH GmbH. (b) Bioprinting of vascularized skeletal muscle. (i) Skeletal muscle structures created by bioprinting are made up of many layers of myofiber bundles in 15 × 15 × 15 mm^3^. Following the removal of sacrificial patterns, microchannels were generated within these structures, preserving the vitality of bioprinted cells. (ii) Immunofluorescent images displaying printed and non-printed samples after 7 d followed by vWF (green) and *α*-SMA (red) in the regenerated tibialis anterior muscles at 4 and 8 weeks post implantation. Reproduced from [200]. CC BY 4.0. (c) Bioprinted prevascularized bone tissue. Computer-aided design representation of a cuboid construct featuring ASCs depicted as brown strands, bioprinted within an osteo-hydrogel using EBB. The ASC strands followed a meandering pattern with a 90° offset between individual layers. A sliced model showing bioprinting of HUVECs represented as red dots, within a fibrinogen matrix using DBB, arranged in the form of grids with three dotted lanes per layer. Thrombin (blue dots) was simultaneously bioprinted as a crosslinker to convert fibrinogen into fibrin and macroscopic image of the bioprinted construct after removal of the PCL frame. (ii) Immunohistochemical staining with human-specific antibody against CD31. Reproduced from [201]. CC BY 4.0. © 2020 The Authors. Biotechnology and Bioengineering published by Wiley Periodicals LLC. (d) Biomanufacturing of the vascularized heart. (i) A diagram illustrating the SWIFT system. (ii) A perfusion system used to evaluate tissue viability after bioprinting. (iii) A series of pictures demonstrating the embedded bioprinting of a branching, hierarchical channel network within a compacted embryoid body-based tissue matrix. Reproduced with permission from [202].© The Authors, some rights reserved; exclusive license AAAS. Distributed under a CC BY-NC 4.0 license. Reprinted with permission from AAAS. (e) 3D Vascularized liver tissue model. (i) Illustration of the multimaterial bioprinting techniques employed in this study. The study utilized multimaterial bioprinting, involving the use of cell (hepatocyte)-laden inks and fugitive inks, to create 3D vascularized hepatic tissue models in an *in-vitro* setting. (ii) Confocal microscopy images demonstrating 3D constructs and cross-sectional views to depict capillary-like networks. [203] John Wiley & Sons. © 2021 Wiley-VCH GmbH.

Several allogeneic multilayered skin grafts have been tested for impaired non-healing cutaneous wounds but such skin grafts have failed over time permanently due to lack of complex skin microvasculature crucial for integration with the host tissue [191]. To meet such requirements, perfusable, vascularized skin substitutes were demonstrated to improve dermal and epidermal maturation [191]. The grafts were fabricated via EBB using two different types of type I collagen-based bioinks: human-derived (1) dermal fibroblasts (FBs), EC, and placental pericytes (PCs) for the dermis layer, and (2) keratinocytes (KCs) for the epidermis layer. After bioprinting, grafts were engrafted onto the skin wound site of C.B-17 SCID/bg mice and *in-vivo* perfusion was confirmed at Week 4 [191]. For a complete xeno-free implantation using immunodeficient (C.B-17 SCID/bg) mice, a bioink comprising of human collagen type I and fibronectin was developed and embedded with cells in the same manner (a dermal layer with EC, FBs, and PCs and an epidermal layer with KCs) [192]. The bioprinted bilayered substitutes revealed skin-specific features like rete ridge-like structures found a mature stratified epidermis and perfusable microvessels within 2 weeks. These studies suggest that the anatomically accurate layers and crosstalk among epithelial and other cells (i.e., KCs) are important, which results in beneficial paracrine effects attained from PCs [193, 194]and maturation on KCs. Another study demonstrated that Zn_2_SiO_4_ (ZS) nanoparticles supported vascularized, innervated skin regeneration (figure 6(a)) [195]. In this regard, 8 week-old BALB/c mice were used to establish a 2nd-degree burn model and transplanted with ZS nanoparticles-incorporated into PCL fibrous scaffolds. The local release of Zn and Si ions was favorable to innervation and re-vascularization, which was illustrated by accelerated wound healing process, endothelial-specific marker (CD31), and neuron-specific marker (PGP 9.5) expressions. Along with vascularization, pigmentation is another important feature of the skin tissue protecting epidermal layers from natural UV radiation and substantially influencing social and phychological well-being of patients [196, 197]. Thereby, reproduction of pigmented and vascularized skin substitutes have been attempted. Among various bioprinting approaches, a robotic platform was introduced, which is a hybrid process combined with EBB, plastic compression, and DBB [198]. For the basal layer, human-derived FBs and ECs were encapsulated in collagen type I-based bioink and bioprinted in a single layer via EBB. The plastic compression was firstly introduced and performed for even distribution of the collagen layer in 3D without affecting cell viability. Lastly, DBB was conducted using human-derived KCs and melanocytes to form a patterned array for the epidermis layer. After transplantation to immunodeficient rats, the bioprinted substitutes showed similar percentage of HMB45^+^ melanocytes to the control foreskin and successfully anastomosed to the vascular plexus of the host. In brief, while recent advances in skin substitutes allowed vascularized, innervated and/or pigmented skin wound healing using multiple cell types, *in-vitro* skin grafts have not yet shown similar maturity to native skin in terms of vascularized dermis or stratum corneum structure and should be improved for developing off-the-shelf clinical products [191, 199].

### 4.2. Vascularized skeletal muscle

Skeletal muscle is a dynamic, heterogeneous, and innervated tissue composed of highly differentiated bundles of muscle fibers [204]. Although muscle fibers have potential to regenerate and self-repair from small injuries, large volume of muscle defects and genetic disorders pose a constant clinical challenge. The considerable progress in the domain of bioprinting and the advent of muscle-specific bioinks have led to successful fabrication of bioprinted vascularized muscle constructs mimicking the mechanical, structural and biochemical features of human muscles, which have been tested *in vivo* [10]. This has been evidenced by 3D implantable muscle constructs which were fabricated using a human primary muscle progenitor cell-laden hydrogel bioink, a sacrificing acellular gelatin, and a supporting PCL frame (figure 6(b)) [200]. *In-vitro* evaluation of the muscle constructs was conducted using myosin heavy chain, which confirmed the maturation. Following *in-vitro* analysis, the bioprinted muscle graft was implanted in rodents with tibialis anterior muscle defects and showed densely packed myofiber-like structures with 82% recovery, von Willebrand factor (vWF^+^) vessels, and neurofilament (NF^+^) nerves. Ngan *et al* developed a bioprinting approach to generate muscle constructs composed of myoblast encapsulated GelMA bioink via photo-crosslinking [205]. Bioprinted GelMA constructs were implanted in nude rats, and spontaneous formation of myofibers was observed. Immunohistochemistry analysis revealed characteristic tissue maturity with sarcomeric striations, where enhanced maturation and well-organized interconnections between ingrowth vessels and nerve axons were observed. In addition, Lee *et al* utilized EBB using skeletal muscle-specific bioink comprising of dECM-methacrylate (MA), polyvinyl alcohol (PVA), and human primary muscle progenitor cells, which allows self-alignment and release of bioactive factors [206]. Owing to synergistic effects of topographical and biochemical cues, the bioprinted substitutes revealed neuromuscular junction characteristics by vWF^+^ vessels, NF^+^ axons, and myosin heavy chain (MHC^+^) myofibers when implanted into rats with a tibialis anterior muscle injury model. Also, a coaxial nozzle was utilized to bioprint human skeletal muscle cells (hSKMs) in the skeletal muscle-based dECM via an inner core nozzle and HUVECs in VdECM via an outer shell nozzle [101]. The core–shell filaments were bioprinted in a parallel manner and transplanted into Sprague–Dawley rats with VML injuries. Interestingly, as compared to hSKM-HUVEC mixed filaments, coaxially-bioprinted group revealed higher muscle weight and number of blood vessels and CD31^+^ cells at Week 4. The compartmentalization of the structure (allocation of different dECM) and cell types induced each cell to fully appreciate its environment and promote myogenesis and angiogenesis efficiently. Furthermore, innvervation was observed in the coaxially bioprinted group with acetylcholine receptors (AChRs^+^) and anti-beta III tubulin (TUJ1^+^) cells. Overall, vascular and neural components, such as endothelial and neural cells and growth factors, can be considered to support muscle cell survival, differentiation, and muscle function.

### 4.3. Vascularized bone

Over the past few decades, there has been an exceedingly huge demand for patient-specific bone grafts to repair bone defects [21]. Recently, a group of researchers has developed cuboid shaped, prevascularized bone tissue constructs with human adipose-derived mesenchymal stem cells (ASCs) and HUVECs (figure 6(c)) [201]. EBB and DBB were used to bioprint the constructs. For bioprinting of HUVECs, fibrinogen with various growth factors, such as VEGF, bFGF, and aprotinin, were prepared. Osteo-hydrogel, containing fibrinogen, gelatin, hyaluronic acid, glycerol, hydroxyapatite, VEGF, bFGF, aprotinin, and osteogenically differentiated ASCs, was prepared. Subcutaneous implantation of these constructs into immunodeficient mice revealed the formation of blood vessels and microvessels of various calibers from the bioprinted HUVECs. A calcified bone matrix was produced by bioprinted ASCs indicating ectopic bone formation. However, the implanted substitutes did not maintain their original shapes because of deformation, and a physical support made of hard polymers or bioceramics can be considered to prevent deformation. Regarding this, a bioceramic bioink consisting of collagen, *β*-tricalcium phosphate (*β*-TCP), human-derived ADSCs, and HUVECs was utilized [207]. Bone substitutes were bioprinted via EBB using the bioink formulation (80% *β*-TCP; ADSC:HUVEC at 7.5:2.5 ratio) opted from *in-vitro* studies, in which a high ratio of *β*-TCP had been proved to activate *in-vitro* osteogenesis of ADSCs [208]. After inserting into a mouse model of posterolateral lumbar spinal fusion, all the index of bone regeneration (i.e. fusion rate, bone volume, bone mineral density, and trabecular thickness and number) significantly improved in *β*-TCP bioprinted substitutes. Also, a significant increment in number of blood vessels and CD31^+^ area was observed. The crosstalk between ADSCs and HUVECs secreted growth factors, chemokines, and cytokines, which enhanced angiogenesis and osteogenesis via tumor necrosis factor alpha and Notch signaling pathways [209, 210]. On the other hand, a chemically-modified nanocomposite bioink was proposed using amine-functionalized copper-doped glass nanoparticles, oxidized alginate, and gelatin to improve biological performance and printability via EBB [211]. This composite bioink was bioprinted with osteosarcoma cells and bone marrow stem cells (BMSCs), which demonstrated high cell viability, rapid spreading, and differentiation owing to the presence of cell-adhesive ligands in the nanoparticle-laden bioink. Without growth factors in an *ex-vivo* environment, copper, known to induce secretion of VEGF from BMSCs [212, 213], promoted ion stimulation leading to both osteogenesis and angiogenesis. Further, a GelMA-based bioink added with BMP-4 and BMSCs was employed to reconstitute osteon-mimetic vascularized bone substitutes via EBB [214]. This osteon-mimetic substitute was designed in an heterogeneous structure consisting of the alternation of PCL and BMSC-laden filament, which was seeded with EPCs. Also, their architectures were altered in terms of canal structures (central medullary, peripheral Haversian, and transverse Volkmann canals), and different canal compositions were implanted into BALB/c nude mice subcutaneously and rabbit lateral femoral condyle. Despite low mechanical properties, the hierarchical structure including all canals revealed the highest percent vascularity and bone regeneration. Also, EPCs and BMSCs mutually promoted osteogenesis and angiogenesis by secreting exosomes and related factors [215–217]. Overall, bone substitutes should have adequate vascularization to support bone regeneration, and more bone microenvironment-related cells, such as osteoclasts and endothelial, neural, and immune cells, should be considered.

### 4.4. Vascularized cardiac tissue

Currently, myocardial infarction and congenital heart disorders are the leading cause of death worldwide [218]. Cardiac tissues are conglomeration of different involuntary muscle types. Due to the inherent limitation of host immune rejection to artificial materials, scaffold-free cardiac tissues were established utilizing a commercially-available spheroid bioprinting platform [8]. Spheroids of cardiomyocytes derived from iPSCs, endothelial cells, and fibroblasts were bioprinted on a needle array using the Kenzan method and assembled into scaffold-free cardiac tissues. Cardiac tissues were then transferred to a chamber to electrically stimulate them, where an increased beating rate was observed in all samples. However, due to the absence of a perfusable vasculature, this approach did not demonstrate scalable tissues. In this regard, Skylar *et al* bioprinted cardiac tissue construct utilizing iPSCs-derived organoids (figure 6(d)) [202]. Perfusable channels were embedded utilizing the sacrificial writing into functional tissue (SWIFT) approach demonstrating its scalability. In another study, 3D bioprinted collagen-based constructs were fabricated utilizing the FRESH approach to rebuild the components of heart [219]. The developed collagen scaffold allowed rapid vascularization of the tissue, while maintaining its mechanical strength. Although fully-functional organs remain a challenge to achieve, FRESH allows to recapitulate the anatomical properties of native tissues. In a recent study, *in-vivo* analysis of a bioprinted cardiac patch, consisting of GelMA-cardiac ECM (cECM) and human c-kit+ progenitor cells (hCPC) has shown improved vascularization and reparative functionality in the right ventricle of a rat heart [220]. Specifically, hCPC-GelMA-cECM revealed improved therapeutic effects in tissue remodeling and cardiac functional outcomes, which was shown by increased right ventricular vessel density, improved cell density, and reduced fibrosis area. However, further tissue-level analysis, such as cell types, cell density, and autologous or allogenic cell sourcing, should be optimized to improve therapeutic capability against right ventricle dysfunction. In another study, a highly elastic bioink with the capacity to induce angiogenesis is formulated using GelMA, human tropoelastin (MeTro), and gelatin for cardiac tissue replacement [221]. For bioprinting cardiovascular tissues via EBB, the bioink was encapsulated with cardiomyocytes (CMs), cardiac fibroblasts (CFs), and HUVECs. More specifically, a heterogeneous lattice construct was bioprinted using a CM/CF-laden bioink and a HUVEC-laden bioink, and its biocompatibility was proven with 85% cell viability during 7 d of culture. In addition, the expression of sarcomeric *α*-actinin and CD31 confirmed a mature stage of muscle differentiation and vascularization, which was also demonstrated by the endothelium barrier function. Notably, beating of cardiac cells were identified at Day 5 post bioprinting, and its coordination improved from 10 to 15 d of culture. Despite achievement of beating cardiac tissues, therapeutic effects required to be assessed using a relevant animal model. To address this, cardiac substitutes were developed using a non-mulberry silk-polyethylene glycol di-methacrylate (PEGDMA)-GelMA conductive bioink containing carbon nanotubes (CNTs) via EBB [222]. For the *in-vitro* study, HUVECs in the conductive bioink were bioprinted and matured in a perfusion bioreactor for 14 d, and their proliferation and maturation were displayed with increased DNA content and CD31 gene expression. Further, when neonatal rat primary cardiomyocytes were seeded, beating was observed on Day 2. For the *in-vivo* study, Sprague–Dawley rats with myocardial infarction sites were implanted with the bioprinted cardiac substitutes and revealed minimal immune response, wound healing, and vascular recruitment. Taken together, investigations in cardiac disease model and healing process should be conducted simultaneously for pre-clinical studies.

### 4.5. Vascularized liver

The liver is the largest gland in the human body and plays a crucial role in metabolism, detoxification, bile production and regulation of electrolytes and water among other functions. It contains several resident cell types, which are tightly arranged in a specific hexagonal block called the hepatic lobule, which is the fundamental building block of the liver [223]. Unfortunately, liver failure remains one of the major causes of mortality in the world including hepatitis (inflammation of the liver tissue), which is among top ten global diseases [224]. Due to its physiological complexity and involved multiple cell types, 3D bioprinting is one of the most feasible options for its regeneration. Further, to induce vascularization in bioprinted constructs, ECs can be incorporated. For example, Kang *et al* used ECs along with hepatic cells and created a hepatic lobule (∼1 mm) array. The developed hepatic lobule contained hepatocytes surrounded and compartmentalized by ECs with a lumen structure mimicking the vascular network of a native structure with higher albumin secretion, urea production, and albumin, and CD31 protein levels, along with greater cytochrome P450 enzyme activity [225]. Pimentel *et al* fabricated thick (∼1 cm) and densely populated (10 million cell/ml) tissue constructs with a 3D four-arm branch network and soft tissue-like (1–10 kPa) stiffness that can be directly perfused on a fluidic platform for a lengthy (>14 d) period of time. The encapsulated HepG2 cells demonstrated proliferation, and the microenvironment’s control caused cells to aggregate in spheroids in particular locations [226]. For drug screening applications, Janani *et al* recapitulated the native liver architecture using (1) a human ADSC-derived hepatocyte-like cell (HLC)-laden ECM-based bioink and (2) a HUVEC/human hepatic stellate cell (HHSC)-laden ECM-based bioink [227]. The liver model comprising HLC/HUVEC/HHSC mimicked native alternate cords of hepatocytes and showed enhanced urea synthesis, albumin production, and cytochrome P450 (CPR) activity over 2 weeks. Further, drug toxicity was assessed in different conditions (i.e., non-hepatotoxicants aspirin and dexamethasone), and a follow-up metabolic evaluation demonstrated the response of DNA concentration, CPR activity, and lactate dehydrogenase activity. These results represented the possibility of clinically-relevant vascularized liver model by a dose-dependent hepatotoxic responses. Moreover, Liu *et al* suggested a vascularized liver platform fabricated via a hybrid system including EBB, polydimethylsiloxane (PDMS) encapsulation, perfusion, and transplantation (figure 6(e)) [203]. To begin, a bioink comprising gelatin/GelMA/fibrin embedded with MSCs and HUVECs was used as a supporting bath after crosslinking. Then, a sacrificial ink comprising gelatin/GelMA/fibrin without cells was used to bioprint perfusable channels and got removed. After seeding HUVECs into channels, PDMS was coated to protect the bioprinted structure and connect PU tubes. Perfusion was performed for 7 d, and the integrated pattern and CD31^+^ HUVECs were observed. To develop a liver model, HepG2/HUVEC/human foreskin fibroblast (HFF) aggregates were used to replace HUVECs in the supporting bath, which expressed albumin after 7 d of perfusion *in vitro*. At last, the perfusable liver platform was connected to the arterial vessel of Sprague–Dawley rats, in artery-to-vein mode. Although the implantation could be maintained for only a week due to thrombus formation, the perfusable liver platform suggested an alternative concept to anastomose with the host vascular network and support liver function. In short, using 3D bioprinting, structural and functional characteristics of liver lobules have been developed; however, simulating hepatocyte functions in accordance with zonal variation of the tissue along with the intricate network of blood vessels is still a challenge. Generation of perfusable vascular networks that carry oxygenated blood to hepatocytes at anatomically appropriate sites would produce biomimetic liver constructs.

Overall, advancements in the realm of bioprinting have enabled the fabrication of musculoskeletal and metabolically highly active functional tissues and organs. It has also expanded its arms in a promising concept of bioprinting directly at the site of injury to increase its effectiveness, which is discussed in the following section.

## 5. IOB: translation of bioprinting from bench to bed side

**Figure 7.**
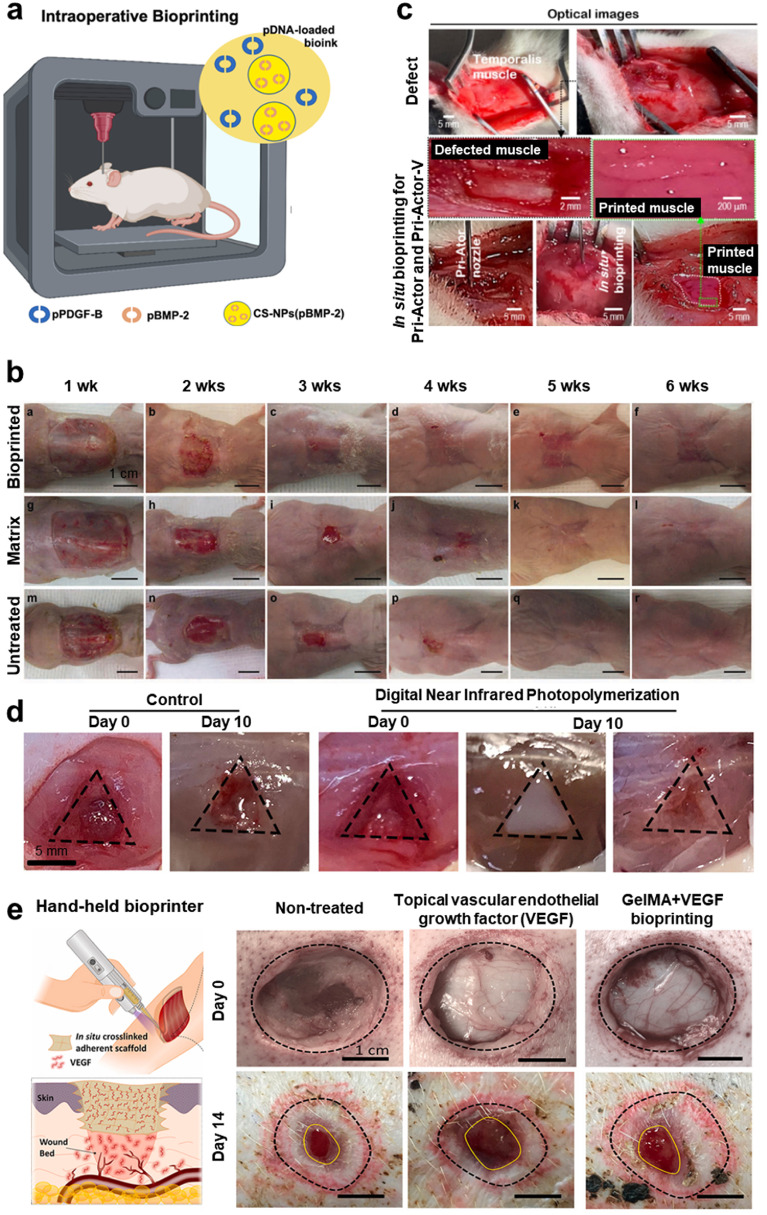
(a) Intraoperative bioprinting (IOB) of bone constructs. This schematic provides an overview of the IOB process for bone regeneration. Reprinted from [229], Copyright (2022), with permission from Elsevier. (b) IOB of autologous skin cells. A time-course evaluation of wound healing in cell-printed mice reveals the early formation of epithelium over the wound by Week 1, followed by the development of skin covering the entire wound by Week 2, although not fully matured. Complete coverage of the wound was observed by Week 3 in cell-printed mice. In contrast, matrix-treated and untreated wounds exhibit minimal epithelialization until Week 4, resulting in a significant proportion of open wound area. Contraction becomes prominent between Weeks 4 and 6 following closure of the wounds. Additionally, a maturing epithelium was observed during this period. Reproduced from [230]. CC BY 4.0. (c) IOB for muscle regeneration. Optical images of a temporalis muscle volumetric loss model in rats and IOB with a pri-actor nozzle. Reprinted from [231], Copyright (2021), with permission from Elsevier. (d) Noninvasive IOB. The digital near-infrared photopolymerization group exhibited faster wound healing compared to the control group. Reproduced with permission from [135]. © The Authors, some rights reserved; exclusive licensee AAAS. Distributed under a CC BY-NC 4.0. Reprinted with permission from AAAS. (e) Handheld Bioprinting. An IOB approach comprises a handheld printer to extrude GelMA precursor mixed with VEGF and *in-situ* photo-crosslinking, which is useful for treating wounds with unusual forms or curved surfaces. *In-situ* crosslinking does not only enable excellent scaffold attachment to the tissue, but it also eliminates the need for additional fixing procedures. The released VEGF promoted angiogenesis inside the wound bed, ultimately improving both the rate and quality of wound healing and representative macroscopic images of wounds subjected to various treatments (topical VEGF, Gelma + VEGF bioprinting) on the 0th and 14th days after surgery. Reproduced with permission from [232]. © 2021 The Authors. Publishing services by Elsevier B.V. on behalf of KeAi Communications Co. Ltd. CC BY-NC-ND 4.0.

One of the significant advancements in the realm of bioprinting in the last decade has been made in IOB, also referred to as *in-vivo* or *in-situ* bioprinting. IOB refers to the process of bioprinting on live subjects in a surgical environment, facilitating the deposition of biologics into a defect site in an anatomically precise and accurate manner (figure 7(a)) [47, 228, 229].

In the literature, IOB was employed for reconstruction of various tissues. For example, using IOB, a healthy skin with dermal structure, extensive deposition of collagen, organized vascular network and proliferating keratinocytes for replacement of extensive wound was illustrated in figure 7(b) [230]. The IOB process was validated in full-thickness excisional wounds on nude mice. Gross examination revealed the appearance of fully-reconstructed skin and faster wound closure at 10–14 d post IOB compared to the untreated defects. Immunohistochemistry showed the presence of keratinocytes and fibroblasts at around 3–6 weeks after IOB. In another study, Moncal *et al* performed IOB to repair skin defects via jetting droplets on Fischer rats [233]. For skin layers, two bioinks were used; the first one was soft-tissue ink (ST-ink), which was made of collagen and fibrinogen loaded with keratinocyte growth factor (KGF) and the second one was the ST-ink loaded with rat primary dermal fibroblasts (rDFs). rDF-laden ST-ink was bioprinted in the dermis layer followed by bioprinting the ST-ink supplemented with KGF in the epidermis layer. At Week 4, the regenerated skin revealed densely packed collagen fibers and blood vessels. Along with histomorphometric characterization, antibody staining of vimentin and loricrin further demonstrated successful bi-layer skin reconstruction. For the treatment of bony defects, on the other hand, a hard-tissue ink (HT-ink) composed of collagen, chitosan, nanohydroxyapatite (nHA), and *β*-Glycerophosphate disodium salt hydrate was introduced [229, 233]. To perform IOB, HT-ink was loaded with rat bone marrow stem cells (rBMSCs) and bioprinted into critical-sized calvarial defects. At Week 6, the defect site revealed up to 80% closure and significantly higher expression of RUNX2 than that of empty defects.

Since IOB involves direct deposition of bioinks at the site of injury, the bioinks must be compatible and suitable for IOB so as to reduce the duration of surgery and rapidly crosslink to maintain the tissue integrity and moist surroundings and physiological temperature. Once the criteria of bioink for IOB are satisfied, IOB can be an effective, scalable tool to repair volumetric muscle loss (VML) and bony defects. In the literature, collagen bioink loaded with human ASCs and HUVECs was utilized to treat VML (figure 7(c)) [231]. Defects on the temporalis muscle of Sprague–Dawley rats were bioprinted with an ASC/HUVEC-laden collagen bioink, which induced the development of myofibers at Week 5. Further, the fibrosis area was significantly reduced in intraoperatively bioprinted constructs compared to manually implanted counterparts.

In another aspect, IOB had been limited to invasive surgeries as it requires the exposure of the site of application. To overcome this limitation, a group of researchers utilized a digital near infrared photopolymerization-based technique enabling noninvasive fabrication of tissue constructs *in vivo* [135]. The technology involves noninvasive bioprinting of subcutaneously injected bioinks into customized tissue constructs, using *ex vivo* radiation of modulated patterned digital near infrared light figure 7(d). Using this technique, an ear-like tissue with chondrification and muscle tissue were obtained, which opened a new avenue for noninvasive IOB.

IOB has seen significant progress in the on-site fabrication of tissue constructs directly within the affected or diseased areas. However, there is a significant gap in the vascularization of these constructs, a critical feature for tissue viability. Vascularization involves creating functional blood vessel networks within bioprinted tissues, typically through specialized bioprinting techniques and angiogenesis-inducing bioinks [234]. The scarcity of research in this area highlights the need for further investigation to develop robust strategies for effective vascularization in tandem with IOB. For instance, IOB was introduced and gradually established using calvarial defect models. As a first attempt, Keriquel *et al* used LBB for bioprinting slurry nHA onto critical size bone defects (4 mm in diameter) of mice [235]. Prior to bioprinting experiments, laser irradiation was proven to be biocompatible with absence of inflammation. Then, the printed nHA recovered bone region for 3 months while empty defects showed no bone formation at any time. In addition to bone defect achievement, the integrity of Dura mater was observed and confirmed with pulsating blood vessels. After checking the feasibility of IOB on revascularization, this concept was further developed and proved. Kérourédan *et al* utilized LBB for evaluating prevascularization and its effects on bone regeneration [236]. For this study, collagen embedded with MSCs and VEGF was used to prefill bone defects. On the top of collagen layer, HUVECs were either seeded or bioprinted via LBB in different patterns for comparison. As vascularization rate (vr) and bone regeneration rate (br) were measured at two months, randomly seeded group (vr = +167%; br = +177%) and LBB group in disc pattern (vr = +203%; br = +294%) and crossed circle pattern (vr = +355%; br = +602.1%) revealed significant differences. These findings denote that *in-situ* prevascularization synergistically promoted both vascular network formation and host tissue (bone) regeneration. Nevertheless, although LBB allows high precision on a micrometer scale, its scalability remains questionable. Further, a clear understanding of anastomoses mechanism is absent, so it remains uncertain whether host vasculature remodeled vascular network or the implanted HUVECs get involved to the angiogenic development and connected with the host vasculature. Clarifying the role of implanted endothelial cells will be helpful to specify their optimal cell density and ratio with respect to other cell types.

Besides, hand-held IOB is emerging as one of strategies for vascular formation. To begin, a hand-held droplet-based bioprinter was presented to achieve (1) contactless, direct bioprinting of cell-laden fibrin or collagen type I and (2) vascular-tube formation via a co-culture of endothelial and dental pulp cells [237]. Specifically, the jetted hydrogels were loaded into root canals in a controlled manner using a miniaturized hand-held bioprinter considered as a simple yet ideal configuration for a future clinical application and dental pulp regeneration. After co-culturing endothelial and dental pulp cells for 14 d, successful vascular tube formation was observed with two-photon microscopy images showing an endothelial-specific marker, CD31, and the longest vascular tube length in 0.5% fibrin followed by 0.3% collagen. However, this study was conducted using bovine teeth, so translation to human teeth is required to test clinical relevancy. In another study, Quint *et al* developed a hand-held extrusion-based bioprinter combined with UV crosslinking system providing thermal insulation of the syringes, rapid exchange of syringes, and fine-tuned continuous extrusion control [238]. These features allowed to bioprint and polymerize a bioink comprising GelMA and VEGF-bind laponite on a VML mouse model. Eight weeks after injury, the number of CD31 positive capillaries was quantified. The increment of CD31^+^ capillaries was significant only in VEGF^+^ bioprinted group owing to the upregulated release of VEGF from both VML and bioprinted substitutes. In parallel, VEGF^+^ group revealed increased hypertrophy in the muscle and reduced fibrosis. The concept of same hand-held bioprinter was further proved using a porcine model with a full-thickness skin wound (diameter = 2.54 cm) (figure 7(e)) [232]. A GelMA bioink with VEGF was used for IOB, and the experimental groups were varied as bioprinting with/without VEGF and topical delivery of VEGF in phosphate buffered saline. When vWF^+^ area was compared on Day 14 post surgery, only VEGF^+^ bioprinted group was significantly higher than the non-treated group. Noteworthy, a sustained delivery of VEGF in bioprinted substitutes was clearly more efficient than the topical delivery of VEGF. Coherently, VEGF^+^ bioprinted group accelerated skin regeneration in terms of wound contraction, epidermal thickness, and rete ridges.

Although notable progress has been made in the realm of IOB for *in-situ* reconstruction of tissues or organs, there is still a plethora of space for improvement to translate IOB from bench to bedside. Especially, revascularization using IOB has been achieved to the limited extent since the goal of IOB has been focusing more on reconstruction of tissues or organs but not solely on vasculature [239]. Although some studies represented vascularized tissues via IOB, indirect vascularization strategies, such as the introduction of angiogenic factors or relying on angiogenic capacity of host vasculature, have been used. These strategies can be oriented from the difficulty of reconstructing on-site perfusable vascular constructs. To overcome this, various existing vascularization strategies can be considered and converged with IOB, such as the addition of ECs or stem cells and surgical procedures (i.e., micropuncture) allowing rapid vessel formation. Shortly, extensive investigations should be followed for reconstructing vascularized, functional tissues and organs via IOB.

## 6. Conclusion

Advancements in the domain of bioprinting over the past decade have significantly expanded its applications in regenerative medicine. Although several tissue grafts have been constructed earlier using bioprinting, they proved to be inadequate due to the absence of vascular interconnections and blood supply, which is crucial for their clinical translation. This review covered bioprinting and its essential steps in the context of vascularization and highlighted the recent progress and achievements made in bioprinting of vascularized tissue and organ substitutes. Despite considerable progress, challenges persist in sourcing angiogenic bioinks and optimizing stem cell-derived endothelial cells. Furthermore, comprehensive translational studies involving anatomically relevant large animal models are necessary. Overcoming these hurdles through rigorous testing, validation, and regulatory processes holds the promise of bioprinted tissues and organs as valuable assets for advancing regenerative medicine and future clinical applications.

## Data Availability

The data that support the findings of this study are available upon reasonable request from the authors.
